# XueBiJing injection reduced mortality in sepsis patients with diabetes

**DOI:** 10.3389/fphar.2025.1413597

**Published:** 2025-02-27

**Authors:** Yan Liu, Hengheng Dai, Yixuan Li, Tianyi Yang, Dandan Zhang, Chaoyue Hu, Si Liu, Zhiqiao Feng, Chi Zhang, Xiaohui Yang

**Affiliations:** ^1^ Beijing University of Chinese Medicine, Beijing, China; ^2^ Key Laboratory of Chinese Internal Medicine of Ministry of Education, Dongzhimen Hospital, Beijing University of Chinese Medicine, Beijing, China; ^3^ Dongzhimen Hospital, Beijing University of Chinese Medicine, Beijing, China; ^4^ Rollins School of Public Health, Emory University, Atlanta, GA, United States; ^5^ Institute for Brain Disorders, Beijing University of Chinese Medicine, Beijing, China; ^6^ Tianjin Chase Sun Pharmaceutical Co., LTD, Tianjin, China

**Keywords:** XueBiJing injection, sepsis, diabetes, pooled data, *post hoc* analysis

## Abstract

**Introduction:**

Sepsis patients with diabetes are at a high clinical risk. It is well reported that XueBiJing injection has good clinical benefit in sepsis individuals. However, there is no relevant report about the efficacy and safety of XBJ in sepsis patients with comorbid diabetes.

**Methods:**

Data of two large randomized controlled clinical trials (XBJ-SAP (ChiCTR-TRC-13003534) and EXIT-SEP (NCT0323874)) were combined, and *post hoc* analyses were performed. Sepsis patients with diabetes were further divided into the XBJ-treated group and placebo group based on inclusion and exclusion criteria. The primary (28-day mortality) and secondary outcomes (mortality in the ICU and in the post-randomization hospital, acute physiology, and chronic health evaluation II (APACHE II) score and sequential organ failure assessment (SOFA) score) were compared between the XBJ treatment and placebo groups in sepsis patients with the diabetes status at baseline. Moreover, the occurrence of adverse events (AEs) was also assessed.

**Results:**

At the study baseline, a total of 378 sepsis patients (227 men [60.0%] and 151 women [40.0%]; mean [SD] age, 60.3 [11.1] years) were considered to have diabetes, of which 177 received XBJ and 201 received placebo administration. Among these sepsis patients with diabetes, the mortality at 28 days was significantly lower in the XBJ group than in the placebo group (29 of 173 patients [16.8%] vs. 56 of 198 patients [28.3%], *P* = 0.01), and the absolute risk difference was 11.5% (95% CI, 3.1%–19.9%). Furthermore, there was no difference in the overall incidence of adverse events (AEs) when XBJ was used (24.4% [42 of 172 patients] vs. 27.7% [54 of 195 patients].

**Discussion:**

The present study underscores the pivotal role of XBJ in modulating the immune response among sepsis patients suffering from diabetes mellitus, exploring the positive effects of XBJ on sepsis patients with diabetes mellitus. The efficacy and safety of XBJ compared with those of the placebo were consistent with the overall trial findings, demonstrating that XBJ is efficacious in sepsis patients with diabetes and suggesting that there is no need for special safety precautions.

**Trial Registration Identifier:**

ChiCTR-TRC-13003534 and NCT0323874.

## 1 Introduction

Sepsis represents a critical health challenge characterized by a disproportionate immune response to infection, leading to high mortality rates among the critically ill patients (Esper and Martin, 2011). Diabetes, one of the most common comorbidities, exacerbates sepsis outcomes, affecting approximately 10%–35% of this patient population (Mayr and Yende, 2016; Schuetz et al., 2011). Diabetes alters the immune system’s response, complicating the management of sepsis due to impaired phagocytosis, cytokine dysregulation, and delayed wound healing (Thimmappa et al., 2023). A recent study examined the relationship between glycemic profiles, disease severity, and outcomes in patients admitted to the intensive care unit (ICU), finding that increased mean blood glucose and glycemic variability were significantly correlated with ICU mortality in sepsis patients, with higher levels elevating the risk of mortality, especially in more severe cases (Lu et al., 2022). Nevertheless, whether diabetes is connected with disease presentation and mortality particularly in sepsis patients remains controversial (van Vught et al., 2016). Over the past decade, the desired curative effect of multiple compounds in clinical trials of severe sepsis has not yet been demonstrated (Opal et al., 2014). In addition, it is crucial to note that effective clinical trial design based on pre-specified subgroups in sepsis patients is limited by the pathogenesis of severe sepsis. Blood glucose level has different effects on disease and is generally not included as a confounder (van Vught et al., 2017). Some findings suggest that pre-existing diabetes increases mortality risk in sepsis patients (Yende et al., 2010; Esper et al., 2009), but others have reported a neutral or reduced risk (de Miguel-Yanes et al., 2015; Koh et al., 2012). It is noteworthy that patients with diabetes tend to be older and have a worse baseline condition. Further evidence is still needed to determine the impact of pre-diabetes on the disease severity of sepsis.

Accumulating evidence supports the efficacy of XueBiJing injection (XBJ) for the sepsis immune-inflammatory response. XBJ is an intravenous preparation derived from traditional Chinese medicine, consisting of extracts from *Carthamus tinctorius L.* (Honghua, HH)*, Salvia miltiorrhiza Bunge* (Danshen, DS)*, Paeonia lactiflora Pall.* (Chishao, CS)*, Ligusticum striatum DC* (Chuanxiong, CX)*,* and *Angelica sinensis* (Oliv.) *Diels* (Danggui, DG). These botanical drugs are known for their anti-inflammatory, antioxidant, and anticoagulant properties, which may synergistically improve sepsis outcomes by modulating the immune response, reducing oxidative stress, and enhancing microcirculation (Li et al., 2021). Recently, two separate large, multicenter, parallel randomized clinical trials have verified that XBJ administration was related with improved survival outcomes in critically ill sepsis patients (Song et al., 2019; Liu et al., 2023). Two separate meta-analyses also provided evidence that XBJ improved 28-day mortality and was associated with good outcomes in sepsis patients (Li et al., 2018; Chen G. et al., 2018). However, outcome data from large confirmatory trials in populations with diabetes and sepsis related to XBJ are lacking.

This study aims to explore the efficacy and safety of administering XBJ injection in sepsis patients with comorbid diabetes, focusing on its immune-modulating effects and contribution in reducing mortality rates.

## 2 Methods

### 2.1 Study design

This study was a *post hoc* analysis that combined data from two clinical trials, XBJ-severe community-acquired pneumonia (SAP; ChiCTR-TRC-13003534) and the efficacy of XBJ injection for sepsis (EXIT-SEP; NCT0323874). Details about the study design have been described before. Each clinical trial protocol was approved by the relevant ethics review committees. The trial (SAP; ChiCTR-TRC-13003534) was approved by the Medical Ethics Committee of Zhongshan Hospital, Fudan University. Participants were enrolled at 33 public tertiary care teaching hospitals in China. This trial (EXIT-SEP; NCT0323874) was approved by the Ethics Committee of Zhongda Hospital, Southeast University (2017ZDSYLL025-P01), and the institutional review board (or independent ethics committee) at each participating site.

This study was approved by the Beijing University of Chinese Medicine Dongzhimen Hospital Ethics Committee (2023DZMEC-294-01), and the trials were performed in compliance with the CONSORT reporting guidelines, the International Conference on Harmonization Guidelines for Good Clinical Practice, and the Declaration of Helsinki and as part of the Efficacy and Effectiveness of Traditional Chinese Medicine (TCM EPLUS) project.

### 2.2 XueBiJing injection

XueBiJing injection, an intravenous preparation, was approved by the former State Food and Drug Administration (CFDA) for treatment of sepsis and multiple organ dysfunction syndrome approved by the National Class II new drug. In April 2020, the Application for Drug Supplement issued by the current National Medical Products Administration (NMPA) approved XBJ injection for the treatment of severe COVID-19, critical systemic inflammatory response syndrome, and multiple organ failure (the official approval document can be found in Supplementary Material). To enhance the accuracy and reproducibility of our research findings, we adhered to the ConPhyMP consensus for reporting traditional Chinese medicine formulas (Heinrich et al., 2022). The scientific nomenclature of the botanical drugs was standardized using the reference by Rivera et al. (2014). Furthermore, we validated the botanical information using the “Plant of the World Online” (http://www.plantsoftheworldonline.org) and “The World Flora Online” databases (WFO, http://www.worldfloraonline.org/). The chemical composition of the drug is available in Supplementary Material, which is provided by the SAP trial (ChiCTR-TRC-13003534).

### 2.3 Participants, randomization, and procedure

Participants were enrolled from two clinical trials. ① XBJ-SAP (*Critical Care Medicine* 2019) included patients aged 18 to 75 years, whose clinical symptoms were suggestive of community-acquired pneumonia and met SAP criteria, which are defined by the American Thoracic Society (Moran et al., 2013). In the XBJ-SAP trial, patients with grade 3.0 sepsis at baseline were not prospectively identified. Grade 3.0 sepsis refers to the criteria defined by the Third International Consensus Definitions for Sepsis and Septic Shock (Sepsis-3), which includes patients with an acute change in the total Sequential Organ Failure Assessment (SOFA) score of 2 points or more due to infection (Singer et al., 2016). However, patients with grade 3.0 sepsis were identified according to the current definition of sepsis, based on reports obtained at the time of randomization. Therefore, it is reasonable to assume that this requirement was met in all enrolled patients. Data were collected during the first 24 h of ICU admission to assist in estimating the proportion of patients diagnosed with sepsis. This trial was conducted with a total of 710 participants with severe community-acquired pneumonia. Using a central randomization system, participants were randomized (1:1) into groups receiving either XBJ or placebo for 5–7 days with a 28-day follow-up. Participants receive 100 mL of XBJ (manufactured by Tianjin Chase Sun Pharmaceutical Co. Ltd., Z20040033) mixed with 100 mL of normal saline every 12 h or matching placebo (200 mL) for 5 consecutive days. These interventions were administered by a dedicated study nurse or a trained ICU nurse. ② Patients with sepsis who were evaluated for admission to the ICU were included in the EXIT-SEP (*JAMA Internal Medicine 2023)*. The sepsis 3.0 criteria (Rivera et al., 2014) are as follows: having an acute change in total SOFA score ≥2 points consequent to the infection. A total of 1,817 subjects who met Sepsis 3.0 criteria were randomized (1:1) to receive either XBJ or placebo. The participants received the solvent only (normal saline, 200 mL, q12 h) in the placebo group, and the solvent plus XBJ (normal saline 100 mL + XBJ 100 mL, q12 h) was administered in the XBJ group. XBJ, specification 10 mL/ampule, packaging 10 ampules/container, at a concentration of 0.1 g/mL, was manufactured by a Good Manufacturing Practice-certified company in China (Tianjin Chase Sun Pharmaceutical Co., Tianjin, China (lot numbers 1304291, 1401091 and, 1501261). Generally, the treatment duration of the study was at least 5 days. All participants received conventional treatment simultaneously. The study protocols, which include a detailed description of the intervention, have been published (Supplementary Material).

The data from both the two studies were monitored by independent monitors, and the data were monitored centrally by staff from the coordinating center according to a prespecified monitoring plan.

A subgroup of patients with diabetes was selected for this analysis based on the following inclusion criteria: (i) a history of type II diabetes mellitus and (ii) baseline medications for diabetes treatment. Sepsis patients with diabetes in XBJ-SAP and EXIT-SEP were further assigned to XBJ or placebo using an interactive web response system. The allocation was determined by a statistician using computer-generated random numbers in a 1:1 ratio.

### 2.4 Outcome measures

In this study, the all-cause mortality at 28 days after randomization was the primary outcome, and secondary outcomes included ICU mortality, inpatient mortality, duration of stay in the ICU and hospital, 28-day non-ICU days (maximum [best] was 28 days; minimum [worst] was 0 days), the cumulative number of days without mechanical ventilation within 28 days, and changes in the SOFA score as well as APACHE II score on days 3 and 6.

The APACHE II score ranges from 0 to 7; the higher score suggests that the more serious the condition, the higher the risk of death. The SOFA score ranges from 0 to 20, with a higher value indicating worse organ function. The change in the SOFA score was based on the score at the time of measurement minus the initial score. Furthermore, the follow-up within 28 days of adverse events (AEs) and serious adverse events (SAEs) was a safety outcome of interest.

### 2.5 Statistical analysis

Data from XBJ-SAP and EXIT-SEP were pooled in this analysis. The statistical analysis plans for XBJ-SAP and EXIT-SEP have been reported in advance. The analysis reported here continued as originally planned.

The generalized linear model with a binomial distribution and identity link function was used to analyze the primary outcomes. Subjects with unknown death status on day 28 were excluded. ICU and hospital mortality also use the same approach. Survival curves from randomization to day 28 were generated by using the Kaplan–Meier method and the log-rank test.

Changes from baseline in the APACHE II score and SOFA value were analyzed with the use of linear mixed-effects models. The fixed effects in this model included baseline data, therapy, visit, and therapy-by-visit interaction. ICU-free days, those without mechanical ventilation, and those involving no hospital stay, the other continuous variables, also use the linear mixed-effects models without baseline adjustment. The adverse event data were provided for descriptive purposes only.

We conducted two sensitivity analyses. Multiple imputations for the missing data were performed under the missing-at-random assumption. Specifically, 100 imputed data sets were generated using the fully conditional specification method, with the number of iterations set to 10 for the following variables: group (XBJ and placebo) and response variable (28-day mortality: yes, no). After multiple imputations, each of the hundred multiple imputation datasets was analyzed by using the generalized linear model. The overall estimates were calculated using Rubin’s rules. Second, to account for baseline imbalances, potential confounders were identified through baseline group comparisons (p < 0.05) and adjusted using the generalized linear model described above. This approach aimed to control for the confounding factors and provide a more accurate assessment of the treatment effects of XBJ on sepsis outcomes in diabetic patients.

The efficacy analysis was based on all participants who were randomized, and the safety analysis data set included participants who received at least one treatment modality. We used R software (version 3.4.1) for analysis. A significance level of 0.05 (two-sided) was considered to be statistically significant. The analytical results should all be considered for the nature of generating hypotheses.

## 3 Results

### 3.1 Study population

**FIGURE 1:**
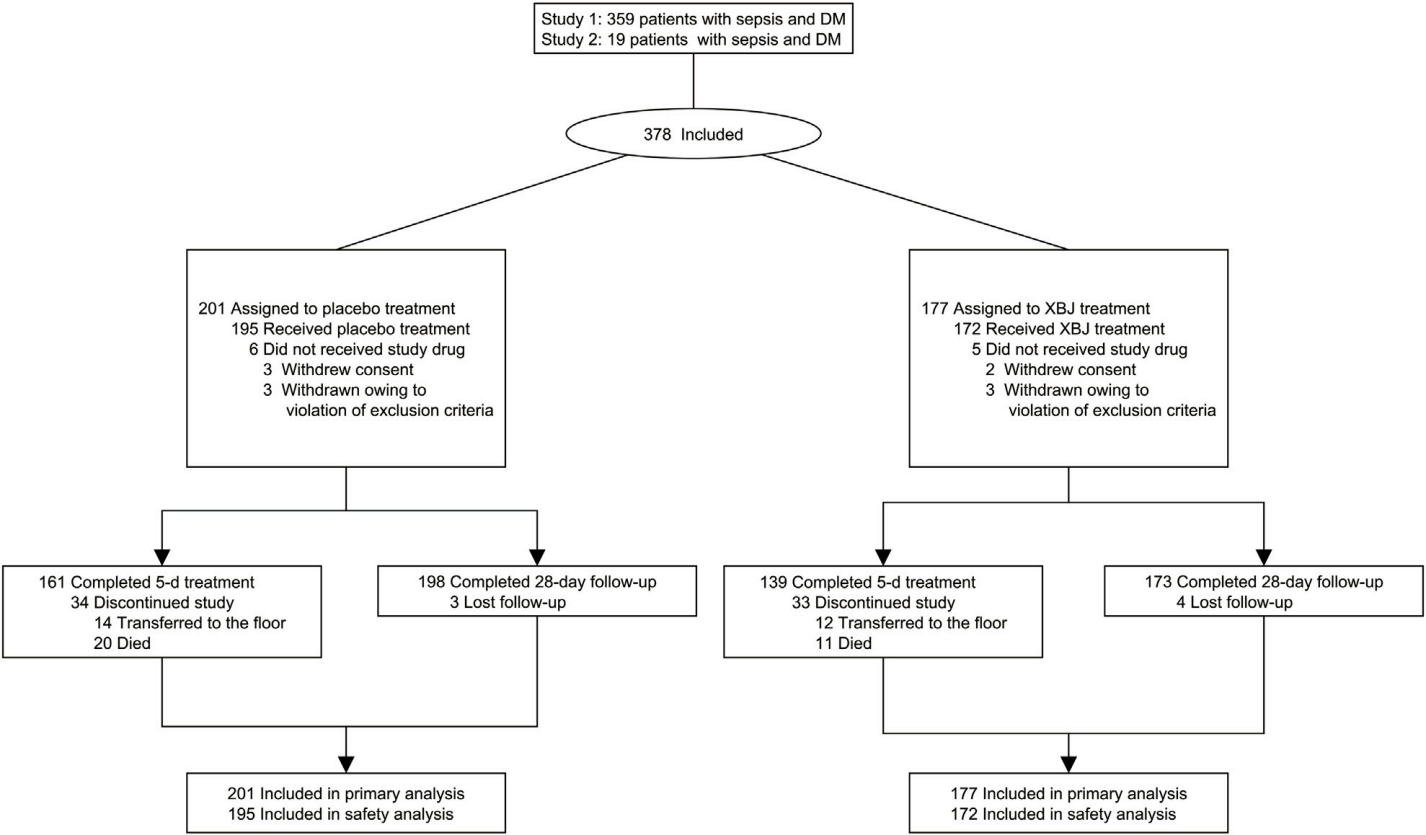
Flow of participants in the efficacy of XueBiJing injection trial.

From September 2013 to July 2019, 2,823 and 4,692 patients were enrolled into XBJ-SAP and EXIT-SEP, respectively. Approximately 53 hospitals in China participated in this study. A total of 2,527 patients were included in the intention-to-treat population. Based on the inclusion and exclusion criteria described above, 378 (15%) of the XBJ-SAP and EXIT-SEP trial participants were found to have diabetes (Figure 1), of whom 19 were in the XBJ-SAP database and 359 were recorded in the EXIT-SEP dataset. Among these 378 patients identified as having sepsis and diabetes, 177 received XBJ and 201 received placebo. Additionally, seven discharged patients were lost to follow-up (4/3; XBJ group/placebo group, respectively), and 28-day mortality was not ascertained.

Patient baseline demographics were well balanced across the groups, except for age; patients in the XBJ group were slightly younger (59.0 ± 11.5 vs. 61.4 ± 10.7). Illness severity and coexisting conditions were also well-balanced across the groups (Table 1). Mean baseline SOFA scores were approximately 7.2–7.4 in both groups. APACHE II scores were approximately 13–14;48.4% (n = 183) of patients had septic shock at enrollment. Among patients with diabetes, the two most common infection sites were the lungs (39.1%) and abdominal cavity (27.8%), and there was no significant difference between the two groups (Table 1).

**TABLE 1:** Patient characteristics at baseline.

Characteristic	Placebo group (N = 201)	XBJ group (N = 177)	P value
Age, mean (SD), y	61.4 (10.7)	59.0 (11.5)	0.03
Sex, No. (%)			0.95
Men	121 (60.2)	106 (59.9)	
Women	80 (39.8)	71 (40.1)	
BMI, mean (SD)^a^	24.0 (2.8)	24.5 (3.4)	0.08
ICU types, No. (%)			0.62
General ICU	151 (75.1)	140 (79.1)	
Emergency ICU	41 (20.4)	33 (18.6)	
Surgical ICU	6 (3.0)	3 (1.7)	
Respiratory ICU	3 (1.5)	1 (0.6)	
Primary site of infection, No. (%)^b^			0.19
Lungs	77 (39.9)	67 (40.4)	
Intra-abdominal	48 (24.9)	57 (34.3)	
Urinary tract	26 (13.5)	18 (10.8)	
Skin or soft tissue	12 (6.2)	6 (3.6)	
Central nervous system	2 (1.0)	4 (2.4)	
Blood	2 (1.0)	2 (1.0)	
Other	26 (13.5)	12 (7.2)	
Source of infection, No. (%)			0.98
Community-acquired	183 (91.0)	161 (91.0)	
Nosocomial	18 (9.0)	16 (9.0)	
SOFA score, mean (SD)^c^	7.4 (3.1)	7.2 (3.0)	0.58
Organ dysfunction, No. (%)^d^			
Respiratory	160 (79.6)	145 (81.9)	0.57
Coagulation	60 (29.9)	52 (29.4)	0.92
Hepatic	36 (17.9)	26 (14.7)	0.40
Cardiovascular	97 (48.3)	79 (44.6)	0.48
Neurologic	74 (36.8)	57 (32.2)	0.35
Renal	37 (18.4)	47 (26.6)	0.06
APACHE II, mean (SD)^e^	14.0 (6.8)	13.1 (6.1)	0.19
<25	184 (94.4)	162 (94.2)	0.94
≥25	11 (5.6)	10 (5.8)	
Heart rate, mean (SD), beats/min	102.8 (21.3)	104.0 (23.6)	0.62
Respiratory rate, mean (SD), breaths/min	22.3 (6.4)	22.2 (6.9)	0.92
Blood pressure, mean (SD), mmHg			
Systolic	120.4 (23.6)	122.3 (24.9)	0.44
Diastolic	67.6 (13.6)	68.3 (16.4)	0.62
Glucose, fasting morning, mmol/L^f^	12.0 (5.5)	13.0 (5.7)	0.11
Medication within 48 h before randomization, No. (%)^f^			
Glucocorticoid	24 (12.4)	15 (9.0)	0.47
Anticoagulant	55 (28.5)	50 (30.1)	0.37
Vasopressor	99 (51.3)	81 (48.8)	0.69
Antimicrobial agents			
Antibacterial agents	189 (96.9)	160 (93.0)	0.08
Antifungal agents	18 (9.2)	14 (8.1)	0.71
Antivirals	11 (5.6)	13 (7.6)	0.46
Septic shock, No. (%)^f^	100 (51.3)	83 (48.3)	0.56
Mechanical ventilation, No. (%)	109 (54.2)	89 (50.3)	0.44
Detected pathogens responsible for sepsis episodes, No. (%)			
Gram-positive	29 (14.4)	22 (12.4)	0.57
Gram-negative	79 (39.3)	59 (33.3)	0.23
Fungal	29 (14.4)	11 (6.2)	0.01
Virials	2 (1.0)	0	0.50

Abbreviations: XBJ, XueBiJing injection; BMI, body mass index; COPD, chronic obstructive pulmonary disease; SOFA, sequential organ failure assessment; APACHE, acute physiology and chronic health evaluation.

^a^ Calculated as weight in kilograms divided by height in meters squared.

^b^ Data on the primary site of infection were available for 187 patients in the placebo group and 161 in the XBJ group; other sites of infection included unknown sources.

^c^ The SOFA score includes sub-scores ranging from 0 to 4 for each of six botanical drugs (respiratory, coagulation, liver, cardiovascular, neurologic, and renal botanical drugs), with higher scores indicating more severe organ dysfunction.

^d^ Organ dysfunctions were defined as a SOFA score of 2 or higher for each of the six botanical drugs.

^e^ APACHE II scores range from 0 to 71; 0 indicates the lowest prediction of mortality, and 71 indicates the highest. Data on APACHE II scores were available for 195 patients in the placebo group and 172 in the XBJ group.

^f^ Data on glucose were available for 192 patients in the placebo group and 168 in the XBJ group; data on glucocorticoids or anticoagulants or vasopressors were available for 193 patients in the placebo group and 166 in the XBJ group; data on antimicrobial agents or septic shock were available for 195 patients in the placebo group and 172 in the XBJ group.

### 3.2 Primary outcomes

**FIGURE 2:**
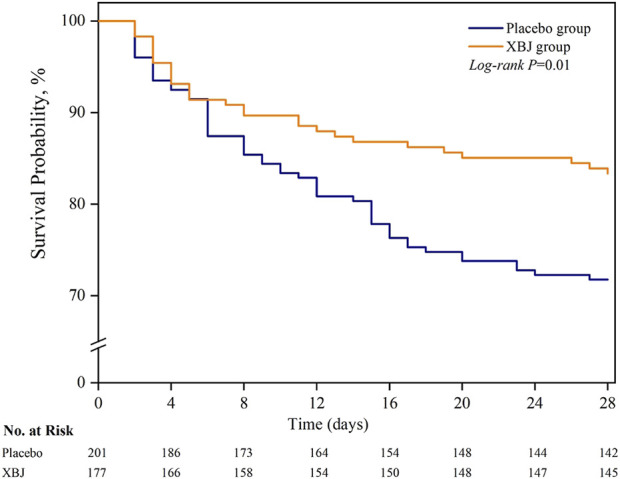
Probability of survival from randomization through day 28. Patients with an unknown survival status at 28 days (n = 5) were censored on the last day they were known to be alive.

Among the sepsis patients with diabetes, 28-day mortality was significantly lower in patients who received XBJ (16.8% vs. 28.3%); absolute difference; 11.5 percentage points; 95% CI 3.1%–19.9% points; P = 0.01) (Table 2; Figure 2). Moreover, we found seven patients were lost to follow-up for survival. On multivariable sensitivity analysis adjusting for age, the results were consistent with the above finding (Supplementary eTable 2.1).

**TABLE 2:** Primary and secondary outcomes^a^.

Variable	Placebo group (N = 201)	XBJ group (N = 177)	Difference (95% CI)	*P* Value
Primary outcome^b^				
28-day mortality, No./total No. (%)	56/198 (28.3)	29/173 (16.8)	11.5 (3.1–19.9)	0.01
Secondary outcomes^c^				
Mortality, No./total No. (%)				
ICU	46/198 (23.2)	25/173 (14.5)	8.8 (0.9–16.7)	0.03
Hospital	49/198 (24.8)	28/173 (16.2)	8.6 (0.4–16.7)	0.04
Length of stay, mean (95% CI), d^d^				
In ICU	10.2 (9.1–11.3)	10.2 (9.1–11.3)	−0.02 (−1.6 to 1.5)	0.98
In-hospital^e^	15.5 (14.2–16.7)	16.6 (15.3–17.9)	−1.1 (−2.9 to 0.7)	0.22
28-day cumulative mechanical ventilation-free days, mean (95% CI), d^f^	17.6 (16.0–19.2)	18.9 (17.2–20.6)	−1.3 (−3.6 to 1.0)	0.28
28-day ICU-free days, mean (95% CI), d^g^	12.5 (11.2–14.0)	14.6 (13.1–16.1)	−2.0 (−4.0 to 0.1)	0.06
Change, SOFA score, mean (95% CI)^h^				
3-day	−1.1 (−1.6 to −0.7)	−1.1 (−1.6 to −0.6)	−0.04 (−0.7 to 0.6)	0.89
6-day	−1.8 (−2.3 to −1.3)	−2.4 (−3.0 to −1.9)	0.6 (−0.1–1.4)	0.09
Change, APACHE II score, mean (95% CI)^h^				
3-day	−2.4 (−3.1 to −1.6)	−2.2 (−3.1 to −1.4)	−0.1 (−1.2 to 1.0)	0.83
6-day	−2.1 (−3.0 to −1.3)	−2.9 (−3.9 to −2.0)	0.8 (−0.5–2.1)	0.24

Abbreviations: XBJ, XueBiJing injection; ICU, intensive care unit; SOFA, sequential organ failure assessment; APACHE, acute physiology and chronic health evaluation.

^a^ For rows including number/total number, the total number refers to the number of patients with valid data.

^b^ A total of seven patients were not documented due to loss to follow-up.

^c^ Missing data not imputed for secondary outcome analyses.

^d^ Data were calculated using a mixed-effects model.

^e^ The length of stay in the hospital included the length of stay in the ICU.

^f^ The mechanical ventilation-free days were defined as the total number of days a patient was alive and not on mechanical ventilation from randomization to 28 days. Data were calculated using a generalized linear model.

^g^ The ICU-free days were defined as the number of days alive and free of ICU stay from randomization to 28 days. Data were calculated using a generalized linear model.

^h^ Negative changes indicate better outcomes. Data were calculated using a repeated-measures mixed-effects model. Data were obtained only from the EXIT-SEP study.

### 3.3 Secondary outcomes

Significant between-group differences were observed in ICU mortality (placebo, 23.2%, vs. XBJ, 14.5%; risk difference, 8.8%; [95% CI, 0.9%–16.7%]; *P* = 0.03) and hospital mortality (placebo, 24.8%, vs XBJ, 16.2%; risk difference, 8.6%; [95% CI, 0.4%–16.7%]; *P* = 0.04). Unlike the EXIT-SEP trial, in the current sub-study, we found no significant differences in ICU-free days and cumulative mechanical ventilation-free days within the 28-day period. There is no significant difference in APACHE II score SOFA score changes after randomization (Table 2).

### 3.4 Adverse event analyses

Overall, there were no differences in the incidence rates of AEs and SAEs between XBJ and placebo in sepsis patients with diabetes (Supplementary eTable 3). In addition, the total frequency of cardiac disorders was higher in patients with diabetes compared to those without diabetes.

### 3.5 Discussion

This pooled analysis of the XBJ-SAP and EXIT-SEP trial yielded two main findings. First, among sepsis patients with diabetes, XBJ was significantly associated with lower 28-day mortality compared with the placebo. This result is consistent with those reported in the XBJ-SAP and EXIT-SEP trials. This may be due to the larger number of patients included and therefore the robustness of the results. After adjustment for cohort and age in clinical trials, the difference remained 10.8 (95% CI, 2.3–19.3) (Supplementary eTable 2.2). Second, XBJ did not increase the risk of AEs in the diabetes subgroup. The present findings suggest that XBJ administration provides benefits to the diabetes subgroup patients, as observed in the XBJ-SAP and EXIT-SEP trials.

The association of sepsis with diabetes mellitus remains a medical issue of considerable importance. A meta-analysis published in 2017 (Wang et al., 2017) evaluated the effect of diabetes on sepsis, finding that the incidence of acute kidney injury (AKI) is significantly higher in septic patients with diabetes mellitus. This underscores the complexity of managing sepsis in diabetic patients and highlights the importance of addressing complications such as AKI. These findings align with our focus on controlled diabetes and its impact on sepsis outcomes. Several studies have found that patients with comorbid diabetes mellitus have a significantly increased risk of AKI (Infante et al., 2022; Kaur et al., 2023). One study analyzed the effect of human recombinant alkaline phosphatase on 7-day creatinine clearance in patients with sepsis-associated AKI. However, compared with the placebo, it did not significantly improve short-term kidney function (Pickkers et al., 2018). Conversely, XBJ administration has been shown to be an effective method for improving the clinical symptoms of sepsis-induced AKI (Yuxi et al., 2017). In addition to sepsis, comorbid diabetes mellitus could cause a fourfold increased risk of death among those developing sepsis (Balintescu et al., 2022). Despite this, the existing studies primarily rely on retrospective cohort analyses focusing on the correlation between metformin use and sepsis outcomes in diabetic patients (Yang et al., 2021; Liang et al., 2019). These studies, however, are limited by small sample sizes and do not conclusively demonstrate that metformin reduces the mortality. Thus, there is an urgent need for high-quality clinical trials to verify the impact of treatments on 28-day mortality. Our study addresses this gap by utilizing data from a clinical trial to investigate the effectiveness of XBJ in reducing mortality among patients with sepsis and diabetes. The XBJ-SAP and EXIT-SEP provided a unique opportunity to assess the 28-day mortality of XBJ injection compared with placebo in critically ill sepsis patients with diabetes as comorbidity. This study first pooled patient-level individual data from two large XBJ randomized clinical trials and suggested that XBJ can be a feasible treatment option for sepsis patients with diabetes.

Diabetes-induced oxidative stress triggers a cascade of immunological dysfunctions. This process releases inflammatory factors that further damage renal function (Jia et al., 2016). XBJ protects against sepsis through multiple mechanisms, including antagonizing endotoxins and inhibiting the release of inflammatory mediators from endotoxin-stimulated cells (Jiang et al., 2013; Li et al., 2019; Chen X. et al., 2018; Liu et al., 2015). It also reduces insulin resistance and improves cell membrane fluidity by regulating cytokine and inflammatory mediator levels, thus mitigating renal microvessel damage caused by hyperglycemia (Jiang and Qu, 2017). Research has shown that XBJ injection can significantly inhibit renal inflammation in septic rats, restore renal microcirculation, and reduce damage to renal tubular epithelial cells (Liu et al., 2021).

Moreover, XBJ’s role in attenuating the hyperglycemia-induced exacerbation of renal microvascular damage further exemplifies its immunotherapeutic benefits. The coagulation and vascular endothelial factors in patients with diabetic nephropathy change with blood glucose levels, influenced by plasma glucose and insulin levels, in turn affecting the body’s coagulation and vascular endothelial function (Ye et al., 2014). Furthermore, disseminated intravascular coagulation (DIC) is a major risk factor for death in sepsis patients. XBJ has been found to help resolve coagulation disorders in DIC (Yin and Li, 2014). Botanical drugs of XBJ, such as ligustrazine and danshensu, inhibit red blood cell and platelet aggregation, improve fibrinolysis, and enhance vascular endothelial function (Gu et al., 2014; Wang and Cao, 2016). Such findings not only underscore XBJ’s utility in addressing the complex interplay between diabetes, sepsis, and immune dysregulation but also highlight the imperative for continued exploration into immune-centric therapeutic strategies within this context. Apart from these effects, XBJ has been shown to inhibit the production of pro-inflammatory cytokines involved in neutrophil recruitment, such as CXCL-1, CXCL-2, and IL-1β, thereby modulating the excessive inflammatory response observed in septic conditions (Wang et al., 2020; Li et al., 2020). Furthermore, its major active components have been found to downregulate GSDMD expression, a key regulator of pyroptosis, in models of depression and cerebral ischemia–reperfusion. Additionally, hydroxysafflor yellow A has been observed to reduce the formation of neutrophil extracellular traps (NETs), which contribute significantly to organ failure and mortality in sepsis (Tian et al., 2021; Tan et al., 2020).

Our study is characterized by several noteworthy limitations. First, the analyses were *post hoc*; therefore, the results presented herein should be considered exploratory. Although previous research has suggested that XBJ exerts its effects through immunomodulation, we did not directly measure inflammatory markers, immune cell profiles, or coagulation parameters in our study. Our findings are primarily based on clinical observations and literature references rather than direct mechanistic validation. Future studies should conduct molecular and cellular experiments to further elucidate the immunoregulatory mechanisms of XBJ in sepsis patients with diabetes. Second, we selected diabetic patients based on their medical history or use of glucose-lowering medications, but the heterogeneity (Yang et al., 2024) within the diabetic population was not fully addressed in this study. We did not stratify patients according to glycemic control or other diabetes-related factors, which could potentially influence the treatment response. Future studies should consider stratifying diabetic patients into subgroups to better understand how these factors impact the efficacy of treatments like XBJ. Third, XBJ-SAP and EXIT-SAP data did not have detailed patient characteristics and diabetes-related factors such as the severity of diabetic kidney disease (DKD) and vascular complications, so we could not clarify the relationship between diabetes and the outcomes. Fourth, rates of adherence and therapeutic changes are not available in these analyses. Fifth, the background therapies investigated herein were not randomized and were thus prescribed based on patient-specific characteristics, prescriber patterns, and regional guidelines and recommendations. Sixth, all study subjects were from China, and the prevalence of diabetes may be different in different regions. Seventh, due to the age limitation of the population included in the original study, we could not include elderly population aged 75 and above, which can also affect the generalizability of the results. Additionally, our baseline data suffered from an imbalance in age distribution. To address this concern and enhance the credibility of our primary analysis, sensitivity analyses were conducted, including age as a covariate. Despite some limitations, the findings are encouraging. In addition, prospective and blinded data collection minimized bias. Moreover, it is the first study to focus on diabetic patients with sepsis. In light of these limitations, it is noteworthy that our findings convey a positive aspect. Despite the acknowledged constraints, the observed outcomes provide valuable insights warranting careful consideration and additional research endeavors.

## 4 Conclusion

This study demonstrates that intravenous infusion of XBJ was associated with a significant reduction in 28-day mortality, and its safety profile was greater than that of the placebo. The efficacy and safety of XBJ compared with those of the placebo were consistent with the overall trial findings.

## Data Availability

The original contributions presented in the study are included in the article/Supplementary Materials; further inquiries can be directed to the corresponding authors

## Abbreviations

95% CI, 95% confidence interval; AEs, adverse events; AKI, acute kidney injury; APACHE, Acute Physiology and Chronic Health Evaluation; BMI, body mass index; COPD, chronic obstructive pulmonary disease; DIC, disseminated intravascular coagulation; DKD, diabetic kidney disease; FPG, fasting plasma glucose; HMGB-1, high-mobility group box 1 protein; ICU, intensive care unit; ITT, intention-to-treat; NMPA, National Medical Products Administration; SAEs, severe adverse events; SAP, severe acute pancreatitis; SD, standard deviation; SOFA, sequential organ failure assessment; XBJ, XueBiJing.
