# Supplementary material for: XueBiJing injection reduced mortality in sepsis patients with diabetes
Source: Front Pharmacol. 2025 Feb 27;16:1413597. doi: 10.3389/fphar.2025.1413597 (PMC11905295; doi:10.3389/fphar.2025.1413597)
Supplement: Supplementary file 2 [file DataSheet4.pdf]

The chemical composition of XueBiJing

ID	Compound	Molecular Mass	Molecular Formula	Average content level
		Da		*μmol/L*
Constituents originating from the component herbs Chuanxiong/Danggui only				
**1**	Senkyunolide I	224.1049	$C_{12}H_{16}O_4$	293.1±28.1
**2**	Senkyunolide H	224.1049	$C_{12}H_{16}O_4$	64.8±6.3
**3**	Senkyunolide G	208.1099	$C_{12}H_{16}O_3$	44.5±3.1
**4**	Senkyunolide N	226.1205	$C_{12}H_{18}O_4$	40.8±5.2
**5**	3-Hydroxy-3-*n*-butylphthalide	206.0943	$C_{12}H_{14}O_3$	37.6±4.8
**6**	*Z*-6,7-Epoxyligustilide	206.0943	$C_{12}H_{14}O_3$	12.7±0.5
**7**	6,7-Dihydroxyligustilide	224.1049	$C_{12}H_{16}O_4$	10.9±1.1
**8**	Senkyunolide A	192.1150	$C_{12}H_{16}O_2$	4.8±2.6
**9**	Senkyunolide J	226.1205	$C_{12}H_{18}O_4$	3.4±0.4
**10**	4-Hydroxy-3-*n*-butylphthalide	206.0943	$C_{12}H_{14}O_3$	2.4±0.3
Constituents originating from the component herb Chishao only				
**11**	Mudanpioside F	344.1471	$C_{16}H_{24}O_8$	5.8±1.0
**12**	1-*O*-β-D-Glucopyranosyl-Paeonisuffrone	360.1420.	$C_{16}H_{24}O_9$	1.6±0.5
**13**	Desbenzoylpaeoniflorin	376.1369	$C_{16}H_{24}O_{10}$	22.1±13.7
**14**	Albiflorin	480.1632	$C_{23}H_{28}O_{11}$	102.4±27.6
**15**	Paeoniflorin	480.1632	$C_{23}H_{28}O_{11}$	2470±142.8
**16**	Oxypaeoniflorin	496.1581	$C_{23}H_{28}O_{12}$	112.9±7.6
**17**	Oxypaeoniflorin isomer	496.1581	$C_{23}H_{28}O_{12}$	4.0±0.8
**18**	Ortho-oxypaeoniflorin	496.1581	$C_{23}H_{28}O_{12}$	4.3±0.7
**19**	Mudanpioside E	526.1686	$C_{24}H_{30}O_{13}$	11.3±1.1
**20**	6′-*O*-Galloyl-desbenzoylpaeoniflorin	528.1479	$C_{23}H_{28}O_{14}$	1.3±0.5
**21**	Benzoylpaeoniflorin	584.1894	$C_{30}H_{32}O_{12}$	58.3±6.8

**22**	Benzoyloxypaeoniflorin	600.1843	$C_{30}H_{32}O_{13}$	1.9±0.2
**23**	Mudanpioside C	600.1843	$C_{30}H_{32}O_{13}$	1.6±0.4
**24**	Mudanpioside J	630.1949	$C_{31}H_{34}O_{14}$	2.0±0.3
**25**	Galloylpaeoniflorin	632.1741	$C_{30}H_{32}O_{15}$	56.0±13.7
**26**	Isomer of galloylpaeoniflorin or galloylalbiflorin	632.1741	$C_{30}H_{32}O_{15}$	7.2±1.5
**27**	Isomer of galloylpaeoniflorin or galloylalbiflorin	632.1741	$C_{30}H_{32}O_{15}$	2.0±0.6
**28**	Galloyloxypaeoniflorin	648.1690	$C_{30}H_{32}O_{16}$	2.4±0.4
Constituents originating from the component herb Danshen only				
**29**	Protocatechuic aldehyde	138.0	$C_7H_6O_3$	139.3±28.7
**30**	Protocatechuic acid	154.0	$C_7H_6O_4$	14.4±1.8
**31**	Tanshinol	198.1	$C_9H_{10}O_5$	66.4±15.5
**32**	Salvianolic acid G	340.1	$C_{18}H_{12}O_7$	0.1±0.0
**33**	Rosmarinic acid	360.1	$C_{18}H_{16}O_8$	40.4±6.3
**34**	Salvianic acid C	378.1	$C_{18}H_{18}O_9$	0.3±0.0
**35**	Salvianic acid C isomer	378.1	$C_{18}H_{18}O_9$	0.3±0.0
**36**	Salvianolic acid D	418.1	$C_{20}H_{18}O_{10}$	0.8±0.3
**37**	Isosalvianolic acid C	492.1	$C_{26}H_{20}O_{10}$	36.0±6.2
**38**	Salvianolic acid C	492.1	$C_{26}H_{20}O_{10}$	3.7±0.9
**39**	Salvianolic acid A	494.1	$C_{26}H_{22}O_{10}$	2.7±0.4
**40**	Salviaflaside	522.1	$C_{24}H_{26}O_{13}$	0.8±0.1
**41**	Lithospermic acid	538.1	$C_{27}H_{22}O_{12}$	1.2±0.2
**42**	Salvianolic acid J	538.1	$C_{27}H_{22}O_{12}$	1.9±0.3
**43**	Salvianolic acid B	718.1	$C_{36}H_{30}O_{16}$	23.9±3.1
**44**	Salvianolic acid E	718.1	$C_{36}H_{30}O_{16}$	1.3±0.3
**45**	4-Methoxylsalvianolic acid B	732.2	$C_{37}H_{32}O_{16}$	0.1±0.0
Constituents originating from the component herb Honghua only				
**46**	Saffloquinoside D	612.169	$C_{27}H_{32}O_{16}$	36.7±3.2

**47**	Saffloquinoside C	612.169	$C_{27}H_{32}O_{16}$	30.0±2.7
**48**	Hydroxysafflor yellow A	612.169	$C_{27}H_{32}O_{16}$	751.2±39.5
**49**	Safflomin C	614.1636	$C_{30}H_{30}O_{14}$	11.3±1.5
**50**	Saffloquinoside E	614.1636	$C_{30}H_{30}O_{14}$	6.7±1.1
**51**	Carthamin	910.2168	$C_{43}H_{42}O_{22}$	0.1±0.0
**52**	Anhydrosafflor yellow B	1044.2747	$C_{48}H_{52}O_{26}$	3.6±1.2
**53**	Kaempferol	286.0477	$C_{15}H_{10}O_{6}$	1.0±0.2
**54**	Kaempferol 3-glucoside	448.1006	$C_{21}H_{20}O_{11}$	9.0±1.3
**55**	Kaempferol 3-rha-(1-6)-glucoside	594.1585	$C_{27}H_{30}O_{15}$	45.6±15.4
**56**	Kaempferol 3-glc-(1-2)-glucoside	610.1534	$C_{27}H_{30}O_{16}$	14.9±2.5
**57**	3-Glc-kaempferol 7-glucuronide or 6-Glc-6-hydroxyapigenin 7-glucuronide	624.1326	$C_{27}H_{28}O_{17}$	12.2±5.6
**58**	6-Hydroxykaempferol 7-glucoside	464.0955	$C_{21}H_{20}O_{12}$	11.2±6.5
**59**	6-Hydroxykaempferol 3-glucoside	464.0955	$C_{21}H_{20}O_{12}$	0.3±0.1
**60**	6-Hydroxykaempferol 3-rha-(1-6)-glucoside	610.1534	$C_{27}H_{30}O_{16}$	3.6±2.0
**61**	6-Hydroxykaempferol 3,6-diglucoside	626.1483	$C_{27}H_{30}O_{17}$	2.2±1.1
**62**	6-Hydroxykaempferol 6,7-diglucoside	626.1483	$C_{27}H_{30}O_{17}$	0.9±0.1
**63**	6-Glc-6-Hydroxykaempferol 3-rha-(1-6)-glucoside	772.2062	$C_{33}H_{40}O_{21}$	9.0±4.2
**64**	6-Hydroxykaempferol 7-(3,6-diglc)-glucoside	788.2011	$C_{33}H_{40}O_{22}$	7.0±2.2
**65**	6-Hydroxykaempferol 7-(3,6-diglc)-glucuronide	802.1804	$C_{33}H_{38}O_{23}$	1.5±0.6
**66**	Quercetin	302.0427	$C_{15}H_{10}O_{7}$	0.7±0.1
**67**	Quercetin 3-glucoside	464.0955	$C_{21}H_{20}O_{12}$	5.6±1.3
**68**	Quercetin 3-rha-(1-6)-glucoside	610.1534	$C_{27}H_{30}O_{16}$	10.0±3.3
**69**	3-Rha-quercetin 7-glucuronide	624.1326	$C_{27}H_{28}O_{17}$	0.7±0.3
**70**	Quercetin 3,7-diglucoside	626.1483	$C_{27}H_{30}O_{17}$	22.5±7.5
**71**	Eriodictyol	288.0634	$C_{15}H_{12}O_{6}$	0.8±0.1

**72**	Neocarthamin	450.1162	$C_{21}H_{22}O_{11}$	10.6±2.1
**73**	Neocarthamin isomer	450.1162	$C_{21}H_{22}O_{11}$	13.8±2.2
**74**	5,6,7,4′-Tetrahydroxyflavone 6,7-diglucoside	612.169	$C_{27}H_{32}O_{16}$	47.3±11.5
**75**	Apigenin	270.0528	$C_{15}H_{10}O_{5}$	0.4±0.1
**76**	6-Hydroxyapigenin	286.0477	$C_{15}H_{10}O_{6}$	0.7±0.2
**77**	Luteolin	286.0477	$C_{15}H_{10}O_{6}$	0.3±0.0
**78**	Luteolin 7-glucoside	448.1006	$C_{21}H_{20}O_{11}$	0.9±0.2
**79**	Scutellarin	462.0798	$C_{21}H_{18}O_{12}$	2.5±1.5
**80**	Safflochalconeside	432.1056	$C_{21}H_{20}O_{10}$	0.1±0.0
Constituents originating from multiple component herbs of XueBiJing				
**81**	p-Coumaric acid	164.0473	$C_{9}H_{8}O_{3}$	69.0±23.1
**82**	4-Glucosyloxybenzoic acid	300.0845	$C_{13}H_{16}O_{8}$	20.9±2.3
**83**	p-Hydroxybenzoic acid	138.0317	$C_{7}H_{6}O_{3}$	52.1±7.0
**84**	Chlorogenic acid	354.0951	$C_{16}H_{18}O_{9}$	13.5±2.3
**85**	Caffeic acid	180.0423	$C_{9}H_{8}O_{4}$	43.2±11.8
**86**	Butanedioc acid	118.0266	$C_{4}H_{6}O_{4}$	118.3±34.0
**87**	Phenylalanine	165.0790	$C_{9}H_{11}NO_{2}$	256.5±28.6
**88**	Uridine	244.0695	$C_{9}H_{12}N_{2}O_{6}$	87.3±13.6
**89**	Cytidine	243.0855	$C_{9}H_{13}N_{3}O_{5}$	1.1±0.1
**90**	Adenosine	267.0968	$C_{10}H_{13}N_{5}O_{4}$	35.5±17.1
**91**	Guanosine	283.0917	$C_{10}H_{13}N_{5}O_{5}$	58.5±10.3
**92**	Adenine	135.0545	$C_{5}H_{5}N_{5}$	23.6±4.6
**93**	Thymine	126.0429	$C_{5}H_{6}N_{2}O_{2}$	3.3±1.4
**94**	Uracil	112.0273	$C_{4}H_{4}N_{2}O_{2}$	48.6±9.8
**95**	benzoic acid	122.0368	$C_{7}H_{6}O_{2}$	767.0±133.9
**96**	gallic acid	170.0215	$C_{7}H_{6}O_{5}$	41.1±15.8
**97**	1'-*O*-benzoylsucrose	446.1424	$C_{19}H_{26}O_{12}$	53.0±24.1
**98**	1'-*O*-galloylsucrose	494.1272	$C_{19}H_{26}O_{15}$	3.2±1.6

**99**	6'-*O*-galloylsucrose	494.1272	$C_{19}H_{26}O_{15}$	3.0±1.6
**100**	Trigalloyl glucose	636.0963	$C_{27}H_{24}O_{18}$	42±17.2
**101**	tetragalloyl glucose	788.1072	$C_{34}H_{28}O_{22}$	8.3±5.1
**102**	tetragalloyl glucose isomer	788.1072	$C_{34}H_{28}O_{22}$	2.5±0.8
**103**	(+)-catechin	290.0790	$C_{15}H_{14}O_{6}$	1.9±2.1
**104**	Ferulic acid	194.0579	$C_{10}H_{10}O_{4}$	117.0±24.4

XueBiJing, an intravenous preparation approved by the China Food and Drug Administration (China FDA) in 2004, has been incorporated into routine sepsis care in China. XueBiJing is prepared from a combination of *Carthamus tinctorius* flowers (Honghua in Chinese), *Paeonia lactiflora* roots (Chishao), *Ligusticum chuanxiong* rhizomes (Chuanxiong), *Angelica sinensis* roots (Danggui), and *Salvia miltiorrhiza* roots (Danshen).

Analysis of chemical composition of XueBiJing was based on liquid chromatography-mass spectrometry by Professor Chuan Li's laboratory at Shanghai Institute of Materia Medica, Chinese Academy of Sciences (Shanghai, China). A part of these results has been published, while details of the others are pending publication elsewhere.

References

(1) Cheng C., Lin J-Z., Li L., Yang J-L., Jia W-W., Huang Y-H., Du F-F., Wang F-Q., Li M-J., Li Y-F., Xu F., Zhang N-T., Olaleye O.E., Sun Y., Li J., Sun C-H., Zhang G-P., Li C. (2016) Pharmacokinetics and disposition of monoterpene glycosides derived from Paeonia lactiflora roots (Chishao) after intravenous dosing of antiseptic XueBiJing injection in human subjects and rats. Acta Pharmacol. Sin. 37: 530–544.

(2) Li X-X., Cheng C., Wang F-Q., Huang Y-H., Jia W-W., Olaleye O.E., Li M-J., Li Y-F., Li C. (2016) Pharmacokinetics of catechols in human subjects intravenously receiving XueBiJing injection, an emerging antiseptic herbal medicine. Drug Metab. Pharmacokinet. 31: 95–98.

(3) Zhang N-T., Cheng C., Olaleye O.E., Sun Y., Li L., Huang Y-H., Du F-F., Yang J-L., Wang F-Q., Shi Y-H., Xu F., Li Y-F., Wen Q., Zhang N-X., Li C. (2018) Pharmacokinetics-based identification of potential therapeutic phthalides from XueBiJing, a Chinese herbal injection used in sepsis management. Drug Metab. Dispos. 46: 823–834.
