# Supplementary material for: XueBiJing injection reduced mortality in sepsis patients with diabetes
Source: Front Pharmacol. 2025 Feb 27;16:1413597. doi: 10.3389/fphar.2025.1413597 (PMC11905295; doi:10.3389/fphar.2025.1413597)
Supplement: Supplementary file 3 [file Table1.docx]

**Herbal-Based Injection Reduced Mortality in Sepsis Patients with Diabetes**

Xiaohui Yang, M.D., PhD, Dongzhimen Hospital, Beijing University of Chinese Medicine, 5 Haiyuncang Street, Dongcheng District, Beijing 100070, China

Tel.&Fax.: +86-10-84013276

E-mail: yxh0616@126.com

Chi Zhang, M.D., Ph.D., Institute for Brain Disorders, Beijing University of Chinese Medicine, Beijing, China

Tel.&Fax.: +86-10-84013209

E-mail: saga618@126.com

eTable 1. Use of Other Medications for Sepsis During Study, N (%) ^a^

|  | Placebo group (N=187) | XBJ group (N=161) |
| --- | --- | --- |
| Glucocorticoid | 47 (25.1) | 30 (18.6) |
| Anticoagulant | 86 (46.0) | 89 (55.3) |
| Vasopressors | 113 (60.4) | 91 (56.5) |
| Antimicrobials |  |  |
| Antibacterial agents | 182 (97.3) | 153 (95.0) |
| Carbapenems | 127 (67.9) | 104 (64.6) |
| Other beta-lactams | 126 (67.4) | 99 (61.5) |
| Quinolones | 47 (25.1) | 36 (22.4) |
| Glycopeptide | 36 (19.3) | 32 (19.9) |
| Oxazolidinones | 40 (21.4) | 26 (16.1) |
| Glycylcyclones | 21 (11.2) | 26 (16.1) |
| Aminoglycoside | 10 (5.3) | 8 (5.0) |
| Macrolide | 1 (0.5) | 2 (1.2) |
| Other ^b^ | 38 (20.3) | 28 (17.4) |
| Antifungal agents | 54 (28.9) | 37 (23.0) |
| Antivirals | 14 (7.5) | 14 (8.7) |

^a^ Medications used during study was only collected for patients receiving the study drug in the placebo group (n=187) and in the XBJ group (n=161) from the EXIT-SEP study.

^b^ Other antibacterial agents included Nitroimidazoles, Sulfanilamide, Polypeptides, Cyclic lipopeptide, Tetracyclines Fosfomycin, Polypeptides, Rifamycin, Anti Mycobacterium drugs, Lincomamides, Chloramphenicols, Furan, Anti tuberculosis drugs, and Rifamycin derivatives.

eTable 2.1. Sensitivity analysis for data missing on the primary outcome

| Variable | Placebo group (N=201) | XBJ group (N=177) | Difference (95%CI) | *P* Value |
| --- | --- | --- | --- | --- |
| 28-day mortality, 95%(CI) | 28.3 (22.0 to 34.6) | 16.8 (11.2 to 22.4) | 11.5 (3.1 to 19.9) | 0.01 |

eTable 2.2. Sensitivity analysis for baseline imbalance (i.e., age) on the primary outcome

| Variable | Placebo group (N=201) | XBJ group (N=177) | Difference (95%CI) | *P* Value |
| --- | --- | --- | --- | --- |
| 28-day mortality, 95%(CI) | 28.0 (21.7 to 34.3) | 17.2 (11.5 to 22.8) | 10.8 (2.3 to 19.3) | 0.01 |

First, we had 7 cases with missing data from the study 1. Multiple imputation was used to impute missing values under the missing-at random assumption (MAR). Specifically, 100 imputed data sets were generated using the fully conditional specification method with the number of iterations set to 10 for the following variables: group (XBJ and Placebo), and response variable (28-day mortality: yes, no). After multiple imputation, each of the hundred multiple imputation datasets was analyzed by generalized linear model. The overall estimates were calculated using Rubin’s rules. The multiple imputation procedure (PROC MI) in SAS, version 9.4 was used (eTable 2.1).

Second, the baseline age was imbalanced from table 1 between groups XBJ and Placebo (59.o vs. 61.4, P=0.03). However, because baseline age is an important prognostic factor, to assess the robustness of the primary analysis, a sensitivity analysis including the age factor as a covariate was performed. The results of unadjusted and adjusted for age showed baseline imbalance in age did not affect the treatment effects (Table 2 and eTable 2.2).

eTable 3. Overall Summary of Adverse Events in Safety Population

|  | Placebo group (N=195) | XBJ group (N=172) |
| --- | --- | --- |
| Any SAE ^a^ | 7 (3.7) | 5 (3.1) |
| Cardiac disorders | 6 (3.2) | 4 (2.5) |
| Atrial fibrillation | 3 (1.6) | 3 (1.9) |
| Myocardial Infarction | 2 (1.1) | 1 (0.6) |
| Arrhythmic storm | 1 (0.5) | 0 (0.0) |
| Gastrointestinal disorders | 1 (0.5) | 1 (0.6) |
| Upper gastrointestinal hemorrhage | 1 (0.5) | 1 (0.6) |
| Any AE | 54 (27.7) | 42 (24.4) |
| Investigations | 50 (25.6) | 41 (23.8) |
| Hemoglobin decreased | 9 (4.6) | 7 (4.1) |
| ALT increased | 6 (3.1) | 8 (4.7) |
| Platelet count decreased | 9 (4.6) | 4 (2.3) |
| Glutamic-oxaloacetic transaminase increased | 7 (3.6) | 4 (2.3) |
| APTT prolonged | 5 (2.6) | 5 (2.9) |
| Bilirubins total increased | 8 (4.1) | 2 (1.2) |
| White blood cell counts increased | 5 (2.6) | 5 (2.9) |
| BUN increased | 4 (2.1) | 5 (2.9) |
| Prothrombin time increased | 2 (1.0) | 5 (2.9) |
| Urine white blood cell increased | 2 (1.0) | 5 (2.9) |
| Blood creatinine decreased | 2 (1.0) | 4 (2.3) |
| Blood creatinine increased | 4 (2.1) | 2 (1.2) |
| Fibrinogen increased | 5 (2.6) | 1 (0.6) |
| Urine red blood cells increased | 3 (1.5) | 3 (1.7) |
| AST decreased | 1 (0.5) | 2 (1.2) |
| Glucose urine elevated | 2 (1.0) | 1 (0.6) |
| Platelet count increased | 1 (0.5) | 2 (1.2) |
| Sugar blood increased | 1 (0.5) | 2 (1.2) |
| Urinary protein increased | 1 (0.5) | 2 (1.2) |
| Decreased white cell count | 0 (0.0) | 2 (1.2) |
| Faecal occult blood positive | 1 (0.5) | 1 (0.6) |
| ALT decreased | 1 (0.5) | 0 (0.0) |
| BUN decreased | 0 (0.0) | 1 (0.6) |
| Blood pressure increased | 0 (0.0) | 1 (0.6) |
| Glutamic-oxaloacetic transaminase | 0 (0.0) | 1 (0.6) |
| White blood cell counts increased | 0 (0.0) | 1 (0.6) |
| General symptoms or Gastrointestinal disorders | 4 (2.1) | 0 (0.0) |
| Fever | 3 (1.5) | 0 (0.0) |
| Pain | 1 (0.5) | 0 (0.0) |
| Musculoskeletal and connective tissue disorders | 1 (0.5) | 0 (0.0) |
| Muscle twitching | 1 (0.5) | 0 (0.0) |
| Respiratory, thoracic, and mediastinal disorders | 0 (0.0) | 1 (0.6) |
| Breathing difficult | 0 (0.0) | 1 (0.6) |

^a^ Data were only from the EXIT-SE
