# Supplementary material for: XueBiJing injection reduced mortality in sepsis patients with diabetes
Source: Front Pharmacol. 2025 Feb 27;16:1413597. doi: 10.3389/fphar.2025.1413597 (PMC11905295; doi:10.3389/fphar.2025.1413597)
Supplement: Supplementary file 4 [file DataSheet3.pdf]

国产药品 ——“国药准字Z20040033”基本信息

批准文号<br>Approval document number	国药准字Z20040033
产品名称<br>Product Name	血必净注射液 Xuebijing injection
英文名称<br>English Name	
商品名<br>Trade Name	
剂型<br>Dosage Form	注射剂 injection
规格<br>Specification	每支装10ml
上市许可持有人<br>Marketing Authorization Holder	天津红日药业股份有限公司 Tianjin Chase Sun Pharmaceutical
上市许可持有人地址<br>Marketing Authorization Holder Address	天津新技术产业园区武清开发区泉发路西
生产单位<br>Manufacturer	天津红日药业股份有限公司 Tianjin Chase Sun Pharmaceutical
批准日期<br>Annotation Date	2021-01-22
生产地址<br>Manufacturing Address	天津新技术产业园区武清开发区泉发路西；天津新技术产业园区武清开发区源泉路17号
产品类别<br>Product Category	中药 Traditional Chinese Medicine
原批准文号<br>Approval Number	国药准字Z20040033
药品本位码<br>Drug Standard Code	86900864000218
药品本位码备注	
注	详情

Table S1. Composition of the Xuebijing injection

Name of Chinese herbal medicine	Botanical plant names	Species	Plant parts used
Xuebijing injection	*Carthamus tinctorius L.*	*Asteraceae*	*Carthamus tinctorius L. flower buds*
	Salvia miltiorrhiza Bunge	*Lamiaceae*	*Salviaemiltiorrhizae radix et rhizoma*
	Angelica sinensis (Oliv.) Diels	*Apiaceae*	*Angelica sinensis (Oliv.) Diels radix et rhizoma*
	Paeonia lactiflora Pall.	*Paeoniaceae*	*Paeonia lactiflora Pall. radix et rhizoma*
	Ligusticum chuanxiong Hort.	*Apiaceae*	*Ligusticum chuanxiong Hort. et rhizoma*
