# Supplementary material for: XueBiJing injection reduced mortality in sepsis patients with diabetes
Source: Front Pharmacol. 2025 Feb 27;16:1413597. doi: 10.3389/fphar.2025.1413597 (PMC11905295; doi:10.3389/fphar.2025.1413597)
Supplement: Supplementary file 5 [file DataSheet1.pdf]

Wang et al. Trials (2016) 17:142
DOI 10.1186/s13063-016-1282-8

Trials

STUDY PROTOCOL Open Access

Xuebijing injection in the treatment of severe pneumonia: study protocol for a randomized controlled trial

Ping Wang[1], Yuanlin Song[2], Zhi Liu[1], Hui Wang[1], Wenke Zheng[1], Si Liu[3], Zhiqiao Feng[3], Jingbo Zhai[1], Chen Yao[4], Ming Ren[1], Chunxue Bai[2*] and Hongcai Shang[1,5*]

Abstract

Background: Severe pneumonia (SP) is a major complication of respiratory system diseases that is associated with high mortality and morbidity. If not treated correctly, it may rapidly lead to sepsis and multiple organ dysfunction syndrome. Despite continuous developments in antibiotic treatments for SP, the mortality rate remains high. Both basic and clinical research show that Xuebijing injection (XBJ) can improve the symptoms of SP. The aim of this study is to evaluate the effectiveness and safety of XBJ compared with placebo.

Methods/design: This multicenter, blinded, randomized controlled trial will be conducted with a total of 700 participants with SP. Using a central randomization system, participants will be randomized (1:1) into groups receiving either XBJ or placebo (within 24 h of diagnosis of SP) for 5–7 days with a 28-day follow-up. All participants will receive conventional treatment simultaneously. Both XBJ and placebo will be administered using a photophobic infusion set to avoid bias. The primary outcome is improvement of Pneumonia Severity Index risk rate. Adverse events will be monitored throughout the trial.

Discussion: This is the first and largest randomized trial done in China on SP treatment using a Chinese herbal extract. In this trial, we will use central randomization and an electronic case report form, and we have designed an innovative blinding method for the traditional Chinese medicine injection. The results of this trial may help to provide evidence-based recommendations to clinicians for treatment of SP.

Trial registration: Chinese Clinical Trials Registry ChiCTR-TRC-13003534. Registered 24 June 2013.

Keywords: Xuebijing injection, Severe pneumonia, Safety, Blinding, Randomized controlled trial

Background

Severe pneumonia (SP) is one of the leading causes of death in patients in the intensive care unit (ICU) [1]. The average mortality in hospitalized patients with SP is between 15 % and 30 %, with a mortality rate of between 50 % and 60 % in the ICU [2, 3]; thus, patients with SP represent a major concern for physicians [4, 5]. If not treated properly, SP may eventually lead to complications that include multiple organ dysfunction syndrome (MODS) and sepsis, which is characterized by persistent inflammation [6], and ultimately death.

Clinical and basic research have revealed that SP is associated with bacterial or viral infections. In response to invasion by pathogenic microorganisms, sustained release of inflammatory mediators leads to systemic inflammatory response syndrome (SIRS) and coagulopathy [7, 8]. Therefore, in addition to active and early treatment for pathogenic microorganisms using antibiotics, anti-inflammatory and anticoagulant therapy may improve the prognosis of patients with SP. Currently, the main therapeutic strategy for SP involves administration of antibiotics and anti-inflammatory agents [9]. However, long-term

* Correspondence: bai.chunxue@zs-hospital.sh.cn; shanghongcai@foxmail.com
[2]Department of Pulmonary Medicine, Zhongshan Hospital, Fudan University, Shanghai Institute of Respiratory Medicine, Shanghai 200032, China
[1]Tianjin University of Traditional Chinese Medicine, 312 Anshanxi Road, Nankai District, Tianjin 300193, China
Full list of author information is available at the end of the article

© 2016 Wang et al. **Open Access** This article is distributed under the terms of the Creative Commons Attribution 4.0 International License (http://creativecommons.org/licenses/by/4.0/), which permits unrestricted use, distribution, and reproduction in any medium, provided you give appropriate credit to the original author(s) and the source, provide a link to the Creative Commons license, and indicate if changes were made. The Creative Commons Public Domain Dedication waiver (http://creativecommons.org/publicdomain/zero/1.0/) applies to the data made available in this article, unless otherwise stated.

antibiotic therapy not only increases the risk of antibiotic resistance but also produces liver and kidney toxicity and other side effects [10]. Moreover, previous anti-inflammatory strategies showed limited efficacy in clinical trials, in part because they targeted single cytokines [11]. Compared with Western medicine, traditional Chinese medicine (TCM) has been shown in recent studies to have promising results for the treatment of SP [12, 13], indicating that, used in complementary and alternative therapies, TCM may represent a novel therapeutic approach for SP.

Wang Jinda, a founder of emergency medicine in China, proposed that integrative medicine therapy can raise the cure rate in acute critical disease [14]. He summarized four rules for four syndromes of intensive and critical care based on prescriptions of a toxin-resolving, blood-quickening decoction (解毒活血汤) and house of blood-expelling decoction (血府逐瘀汤) described by Wang Qingren, a famous physician of the Qing dynasty [8]. Through pharmacodynamic screening, he developed the Xuebijing (XBJ) injection [15], a formula composed of five medicinal herbs. Safflower (*hong hua*) acts as the sovereign drug, activating blood circulation and removing blood stasis, while red peony root (*chi shao*) and *Ligusticum wallichii* (*chuang xiong*) function as the minister drug, cooling blood, dispersing blood stasis, and detoxifying and magnifying the effects of the sovereign drug to activate blood and move qi. *Salvia miltiorrhiza* (*dan shen*) and *Angelica sinensis* (*dang gui*) are assistants which enrich blood and disperse stasis.

In the TCM perspective, the basic pathogenesis of SP is blood stasis and toxicity blockade. XBJ, a Chinese patent medicine for the symptomatic treatment of SP, has the effect of promoting blood circulation and removing blood stasis, as well as mediating fever attenuation and detoxification. The main components of XBJ are hydroxysafflor yellow A, paeoniflorin, ferulic acid, and salvianolic acid B. Basic research has confirmed that XBJ functions as an endotoxin antagonist, an anti-inflammatory agent, and an anticoagulant and that it regulates immune function [16–19]. Previous clinical studies showed that routine medication combined with XBJ may reduce infection indicators and the levels of inflammatory cytokines induced by SP [20]. The present clinical study is associated with limitations of sample size, clinical treatment time, and nonuniform drug dosage, as well as with inconsistencies in inclusion and exclusion criteria. Therefore, a large-scale, multicenter, blinded, randomized clinical trial (RCT) is required to confirm the efficacy and safety of XBJ injection for the treatment of SP.

The aim of this trial is to evaluate the effectiveness and safety of XBJ injection for SP in China by comparison with a placebo.

Methods/design

Research type

We are conducting a randomized, controlled, blinded, multicenter trial.

Study setting

The hospitals enrolled in this study are all tertiary referral medical centers, including 28 Western medicine hospitals and 2 TCM hospitals. All of these hospitals are listed in Table 1.

Study criteria

The patients enrolled in this study should meet the diagnostic and inclusion criteria and provide written informed consent.

Diagnostic criteria

The diagnostic criteria we will use for SP are based on the Infectious Diseases Society of America/American Thoracic Society guidelines [21]. The criteria for SIRS are based on the 1991 Chicago meeting standards [22].

Inclusion criteria

The inclusion criteria are as follows:

1. Age ≥18 years and ≤75 years, male or female
2. Weight ≥40 kg and ≤100 kg
3. Meet the diagnostic criteria for SP
4. Meet the diagnostic criteria for SIRS
5. Provide signed informed consent

Exclusion criteria

The exclusion criteria are as follows:

1. Diagnosis of SP for more than 48 h
2. Pregnant and lactating women
3. Disorders likely to have serious effects on survival of the primary disease (such as unresectable tumors, blood diseases, prolonged bed rest caused by cerebrovascular diseases, Alzheimer's disease, or HIV)
4. Using immunosuppressants, hormones (a cumulative total methylprednisolone dose ≥1500 mg), and/or using cytotoxic drugs within the previous 6 months, or using all of these drug types within the previous 7 days
5. Pneumonitis, interstitial pulmonary fibrosis, alveolar proteinosis, and allergic alveolitis induced by obstructive lung tumors
6. Psychiatric patients
7. Allergies (to two or more substance allergies)
8. Participation in other clinical trials in the previous 30 days
9. Using prohibited drugs in the 7 days before enrollment

Table 1 Research settings and name of each ethics committee

Research setting	Ethics committee name	Approval registration number
Zhongshan Hospital, Fudan University	Medical ethics committee of Zhongshan Hospital, Fudan University	2011-(38)3
Second Military Medical University, Changhai Hospital	Medical ethics committee of Second Military Medical University, Changhai Hospital	CHEC2013-107
First Teaching Hospital of Tianjin University of Traditional Chinese Medicine	Medical ethics committee of First Teaching Hospital of Tianjin University of Traditional Chinese Medicine	SLK2012012-1
Tianjin Medical University General Hospital	Drug ethics committee of Tianjin Medical University General Hospital	IRB2013-021-02
People's Liberation Army General Hospital of Shenyang Military Region	Medical ethics committee of People's Liberation Army General Hospital of Shenyang Military Region	k2013-02
First Affiliated Hospital of Dalian Medical University	Ethics committee of First Affiliated Hospital of Dalian Medical University	LCSY2014-02
Second Affiliated Hospital of Harbin Medical University	Medical ethics committee of Second Affiliated Hospital of Harbin Medical University	2013-研-012
Chinese People's Liberation Army General Hospital	Medical ethics committee of Chinese People's Liberation Army General Hospital	C2014-001-01
First Affiliated Hospital of Chinese People's Liberation Army General Hospital	Medical ethics committee of First Affiliated Hospital of Chinese People's Liberation Army General Hospital	C2014-001-01
Qilu Hospital of Shandong University	National drug clinical trial ethics committee of Qilu Hospital of Shandong University	2013068(1)
Second Affiliated Hospital of Hebei Medical University	Medical ethics committee of Second Affiliated Hospital of Hebei Medical University	2013EC10-01-1
Beijing Union Medical College Hospital, Chinese Academy of Medical Sciences	Ethical review committee of Beijing Union Medical College Hospital, Chinese Academy of Medical Sciences	S-519
People's Liberation Army Air Force General Hospital	Ethics committee of People's Liberation Army Air Force General Hospital	空总药字第2013-04
People's Liberation Army Second Artillery General Hospital	Ethics committee of People's Liberation Army Second Artillery General Hospital	2013013
People's Hospital of Wuhan University	Medical ethics committee of People's Hospital of Wuhan University	[2013]伦审字(066)号
Zhongnan Hospital of Wuhan University	Medical ethics committee of Zhongnan Hospital of Wuhan University	(2013)伦审字(024)号
Wuhan General Hospital of Guangzhou Military Region, People's Liberation Army	Medical ethics committee of Wuhan General Hospital of Guangzhou Military Region, People's Liberation Army	[2013]005-2号
The First Hospital of Jilin University	Ethics committee of The First Hospital of Jilin University	(2014)临审第(140512-049)号
Xin Hua Hospital affiliated with Shanghai Jiaotong University School of Medicine	Medical ethics committee of Xin Hua Hospital affiliated with Shanghai Jiaotong University School of Medicine	XHEC-A-2013-041-2
People's Liberation Army Guangzhou Military General Hospital	Institutional review board for clinical trials of People's Liberation Army Guangzhou Military General Hospital	批件(2014)-0014
West China Hospital of Sichuan University	Clinical test and biomedical ethics branch of West China Hospital of Sichuan University	2013年临床试验(上市)审(14)号
Hunan Provincial People's Hospital	Medical ethics committee of Hunan Provincial People's Hospital	[2015]-06
Beijing Millennium Monument Hospital	Medical ethics committee of Beijing Millennium Monument Hospital	(2013)伦审第(57)号
People's Liberation Army Beijing Military General Hospital	Institutional review board for clinical trials of People's Liberation Army Beijing Military General Hospital	BZEC2013-063
Shiyan Taihe Hospital	Drug clinical trials ethics committee of Shiyan Taihe Hospital	2015第(01)号
Yuhuangding Hospital Affiliated with Qingdao University	Ethics committee of Yuhuangding Hospital Affiliated with Qingdao University	烟毓医伦理审[2014]32号
Guangdong Province Chinese Medicine Hospital	Ethics committee of Guangdong Province Chinese Medicine Hospital	广东省中医院伦理委员会B2013-165-01

Table 1 Research settings and name of each ethics committee *(Continued)*

First Affiliated Hospital of Fourth Military Medical University	Drug clinical trials ethics committee of First Affiliated Hospital of Fourth Military Medical University	KY20140813-3
The First Affiliated Hospital of Anhui Medical University	Clinical trial ethics committee of The First Affiliated Hospital of Anhui Medical University	安医一附院伦审-PJ2015-02-07
The Fourth Affiliated Hospital of China Medical University	Medical ethics committee of The Fourth Affiliated Hospital of China Medical University	2015-020

10. Patients diagnosed with severe acute respiratory distress syndrome [23]
11. Patients who were unsuitable for participation in this trial or unable to complete the follow-up according to the judgment of the investigators

Other criteria

Forbidden and concomitant drugs The following criteria apply to forbidden and concomitant drugs:

1. Use of prohibited medicines, including ulinastatin as well as TCM injections with efficacy similar to that of XBJ, such as Tanreqing and Reduning
2. Details of any additional drugs or therapy must be recorded in the case report form (CRF), including the drug name, dose, and treatment duration

Suspension criteria The criteria for suspension of participation are as follows:

1. Poor compliance of investigators or patients
2. Occurrence of serious adverse events (AEs), complications, or fatal physiological changes
3. Using forbidden medications or treatments during the trial that might affect analysis of the results
4. Voluntary withdrawal
5. Incomplete data
6. Withdrawal for various reasons, such as death or failure to attend follow-up visits

Interventions

Methods of administration

The treatment group receives routine medication [24] plus XBJ injection (specification 10 ml/piece, packaging 10 pieces/container). The control (placebo) group receives routine medication plus a 0.9 % sodium chloride injection. The dosage and speed of injection for the placebo are identical to the XBJ injection (100 ml twice daily, intravenous drip for more than 80 minutes).

Routine medications for SP

The routine medications used in this trial for SP are as follows:

1. *Types of antibiotics*: A β-lactam (cefotaxime, ceftriaxone, or ampicillin/sulbactam) plus either a macrolide (azithromycin, clarithromycin, or erythromycin) or a fluoroquinolone (for penicillin-allergic patients, a respiratory fluoroquinolone and aztreonam); for *Pseudomonas* infection, a β-lactam (piperacillin/tazobactam, cefepime, imipenem, or meropenem) plus either ciprofloxacin or levofloxacin; or the above β-lactam plus an aminoglycoside and an antipneumococcal fluoroquinolone (for penicillin-allergic patients, substitute aztreonam for the above β-lactam)
2. *Dosage of hormones*: Using hormones is one of our exclusion criteria; however, during the intervention period (5–7 days), low-dose (1 mg/kg/day), methylprednisolone can be given if necessary

Dispensing and combination methods

The dispensing and combination methods used in this trial are as follows:

1. XBJ (100 ml) is diluted to 200 ml using saline as a solvent.
2. The use of other injections simultaneously during the course of intravenous infusions is prohibited.
3. Other injections should be separated by injection of 50 ml of saline.

Precautions

The precautions we will observe are as follows:

1. Patients receive the study drug within 24 h of enrollment.
2. The routine treatment of SP (e.g., antibiotics, hormones, anticoagulants, vasopressors) should be carried out simultaneously and XBJ injection should not be used as a substitute.

Sample size

Previous studies [25, 26] showed that the improvement of PSI risk rating of the control group is 70 %. Assuming that the improvement in the treatment group is 10 % higher than that in the control group, the sample size is calculated according to the parameters $\alpha = 0.05$ (two-sided test) and $\beta = 0.2$. Using PASS 11 software (NCSS Statistical Software, Kaysville, UT, USA), we calculated that 291 patients should be recruited into each group. Considering an attrition rate of no more than 15 %, the eligible participants in each group should be no fewer than 342. Therefore, we determined that we would need a sample size of 350 in each group ($n = 700$).

Randomization

The randomization of the trial will be completed at an independent data center using a central randomization system to achieve dynamic minimization randomization. When a subcenter accepts an eligible participant, researchers will log into the central randomization system to enter stratified factor information of the patient, including age, mechanical ventilation condition, and the source of infection (community- or hospital-acquired infection). The central randomization system will then assign an identification code and a random number unique to this participant, who then will receive the corresponding treatment regimen.

Blinding

Blinding methods

The blinding methods used in this trial are as follows:

1. Investigators and drug administrators have independent authority to log into the central randomization system. The investigators are responsible for screening subjects, obtaining informed consent, entering patients' information into the system, and obtaining a random number; however, the patients' group assignment is concealed. After the subjects are randomly assigned by the investigators, drug administrators will log into the system to obtain the patient's group with the random number and assign the study drugs to subjects.
2. Both the XBJ and placebo are administered using the photophobic infusion set to avoid the subjects' ascertaining their group assignment.
3. Both the paper and electronic CRFs are filled in with the patient's random number only, while details of the group information are not included.
4. During the course of the study, investigators and drug administrators work relatively independently, both having signed a confidentiality agreement that prohibits any disclosure of group-related information.
5. Unblinding is divided into two processes by the interactive web response system (IWRS) central randomization system. First, after data-locking, the random numbers corresponding to the group code (e.g., A group, B group) are revealed. Second, when the statistical report is fixed, the actual group will be revealed.

Emergency unblinding

If knowledge of the patient's group is required in the event of an emergency or a requirement for rescue, researchers first obtain details of the patient's group from the drug administrators, then the reason for the unblinding will be reported to the major investigators within 24 h. The subjects are withdrawn from the study after unblinding. Detailed unblinding cause, date, treatment situation, and results will be reported in the CRF and signed by the administrator.

Content and points of data capture

The content and points of data capture in the trial are as follows:

1. Screening period (1 day): 24 h before recruitment
2. Intervention period (5–7 days): follow-up every day and recorded
3. Period after intervention (within 28 days after treatment): follow-up at day 8 and day 28

Different items are measured according to the time points of data collection. The details are shown in Table 2.

Outcome measures

Primary outcome measures

The primary outcome of this trial is the improvement of the PSI risk rating [27]. The evaluation criteria are as follows:

1. *Significantly effective*: the risk rating decreases two levels
2. *Effective*: the risk rating decreases one level
3. *Ineffective*: no change or deterioration in the risk rating

Evaluation of improvement in PSI risk rating is the sum of the total numbers of significantly effective and effective divided by the total number, multiplied by 100 %.

Secondary outcome measures

The secondary outcome measures are as follows:

Table 2 Content and points of data capture

Content	Screening	Intervention							After intervention	
	Visit 1	Visit 2	Visit 3	Visit 4	Visit 5	Visit 6	Visit 7	Visit 8	Visit 9	Visit 10
Day	0	1	2	3	4	5	6	7	8	28
Informed consent form	X									
Pregnancy test	X									
Demographic information	X									
Inclusion/exclusion criteria	X									
Get SSID and random number	X									
History of SP and treatment	X									
History of medication and treatment	X									
Concomitant diseases	X									
Complications	X	X	X	X	X	X	X	X	X	
Body weight and body mass index	X								X	
Vital signs	X	X	X	X	X	X	X	X	X	
Laboratory tests	X				X				X	
Safety outcomes	X				X				X	X
Mean arterial pressure	X	X	X	X	X	X	X	X	X	
Central venous pressure	X	X	X	X	X	X	X	X	X	
Blood gas analysis	X	X	X	X	X	X	X	X	X	
Mechanical ventilation parameters	X	X	X	X	X	X	X	X	X	
Chest x-ray or CT scan	X				X				X	
Sputum and blood culture	X	X	X	X	X	X	X	X	X	X
Record of routine treatment		X	X	X	X	X	X	X		
Issue study drug		X	X	X	X	X	X	X		
Record issue and recovery drug		X	X	X	X	X	X	X		
Record adverse events		X	X	X	X	X	X	X		
Severity-of-illness scores	X				X				X	
Stage efficacy evaluation					X				X	
Length of mechanical ventilation	X	X	X	X	X	X	X	X	X	
Length of stay in ICU	X	X	X	X	X	X	X	X	X	
Length of hospitalization	X	X	X	X	X	X	X	X	X	
Length of antibiotic use	X	X	X	X	X	X	X	X	X	
Efficacy and safety evaluation									X	
Main treatment affecting prognosis										X
Survival follow-up										X

CT computed tomography, *ICU* intensive care unit, *SP* severe pneumonia

1. *SIRS improvement*: determined according to changes in the SIRS diagnostic indicators [28] before and after the intervention. These evaluation criteria are as follows:
   a. *Significantly effective*: the symptoms improved (three or four diagnostic indicators change from abnormal to normal)
   b. *Effective*: the symptoms were relieved (two diagnostic indicators change from abnormal to normal)
   c. *Ineffective*: no change or deterioration in the symptoms (only one diagnostic indicator changes from abnormal to normal, or other condition)
   d. SIRS improvement degree is measured as the sum of the total numbers of significantly effective and effective divided by the total number, multiplied 100 %
2. *Lung Injury Score (LIS) improvement*: only for patients with ventilation; the evaluation criteria are based on those defined by Murray and colleagues

Table 3 Evaluation criteria of Lung Injury Score improvement

Total score of four items	Total scores for the three items (except lung compliance)			
	Decrease ≥4	Decrease 2–3	Decrease 1	No change or increase
Decrease ≥6	Significantly effective	Significantly effective	Significantly effective	Significantly effective
Decrease 4–5	Significantly effective	Moderately effective	Slightly effective	Slightly effective
Decrease 2–3	Slightly effective	Slightly effective	Slightly effective	Slightly effective
No change or increase	Ineffective	Ineffective	Ineffective	Ineffective

[29] according to the changes in LIS before and after the intervention (Table 3), measured as LIS improvement degree equals the sum of significantly, moderately, and slightly effective divided by the total number, multiplied by 100 %

3. The highest body temperature
4. MODS score [30, 31] improvement according to the differences in MODS scores before and after the intervention:
   a. *Significantly effective*: MODS score decrease ≥7
   b. *Moderately effective*: MODS score decrease 4–6
   c. *Slightly effective*: MODS score decrease 2–3
   d. *Ineffective*: no change or increase in MODS score
   e. MODS score improvement degree is measured as the sum of significantly, moderately, and slightly effective divided by the total number, multiplied by 100 %
5. Acute Physiology and Chronic Health Evaluation II (APACHE II) [32] score improvement according to the differences in the APACHE II scores before and after the intervention using the lowest APACHE II score within 24 h (≤19, low risk; ≥20, high risk)
6. Sequential Organ Failure Assessment [33] score improvement assessed according to the number of organ failures (0–1 organ failure, low risk; ≥2 organ failures, high risk)
7. The changes in inflammation and coagulation indicators: C-reactive protein, procalcitonin, and D-dimer
8. Chest x-ray changes
9. The mutual conversion rates of invasive mechanical ventilation and noninvasive mechanical ventilation
10. Mortality rate after 28 days
11. The time of mechanical ventilation, total duration of ICU stay, hospitalization, and antibiotic use
12. The time of bacterial cultures becoming negative

Safety outcomes

Safety outcomes, including vital signs, routine blood and urine tests, fecal occult blood test, hepatic (alanine transaminase, aspartate transaminase, serum total bilirubin) and renal (blood urea nitrogen, creatinine) function, coagulation index (fibrinogen, prothrombin time, activated partial thromboplastin time), electrocardiogram results, and AEs. All of these indicators will be monitored closely throughout the trial.

Adverse events

Every AE occurring during the study must be recorded in the AE form according to the actual circumstances. The following information should be recorded: occurrence time, severity, duration, adopted measure, and the outcome of the AE. The number and rate of AEs and serious AEs of the two groups are recorded. A crossover table is used to describe the changes in laboratory and electrocardiogram indicators.

Statistical analysis

The statistical analysis will be performed using SAS software version 9.4 (SAS Institute, Cary, NC, USA). Rates of PSI improvement between the groups will be analyzed by performing superiority tests. If the lower limit of the 95 % confidence interval is larger than a clinically meaningful difference, therapeutic effects of the experimental group are deemed to be clinically and statistically better than those of the control group. Two-sided tests will be performed for all the other statistical analyses. Cochran-Mantel-Haenszel χ^2 tests or Fisher's exact tests will be used for comparison of categorical outcomes. Continuous outcomes will be analyzed by using Student's *t* test. *p* values 0.05 are considered to indicate statistical significance.

Data management

Data input

Two data input media will be applied in this study. Researchers add patient information to the paper CRF promptly and synchronously with input into the electronic CRF. The occurrence of unexpected problems during this process should be recorded, and the data management center will be informed in a timely manner.

Data verification

In this trial, Oracle Clinical (OC) software (Oracle Corp., Redwood Shores, CA, USA) will be used for centralized data management [34]. Modifications made by clinical investigators will be checked promptly, and the results will be reported to the researchers and clinical research

associates (CRAs). The CRAs are responsible for verifying the consistency and accuracy of the paper and electronic CRFs and for reporting the results to the clinical investigators.

Data lockup

Data lockup will be implemented by data management on completion of the study. Researchers are unable to modify data subsequently, and problems will modified in the statistical analysis.

Quality assurance

Compliance control

Before the trial, caution will be applied in selection of the participating institutions and investigators. All the participating institutions will be required to have approval from the drug clinical trial agency, and all the investigators will be required to be qualified in the implementation of Good Clinical Practice (GCP) training according to the State Food and Drug Administration (SFDA). Before the trial, investigators will receive rigorous training and take a comprehensive examination to improve compliance [35]. In the CRF, the investigators will be required to provide authentic and reliable data of combined medication and AE conditions; the subjects will be required to comply with their medication regimen and receive follow-up in accordance with the trial plan; and the drug administrator will ensure accurate recording of the dosage and amount of drug remaining to monitor patient compliance.

Monitoring and inspection

Both online monitoring and in situ supervision will be used in this trial. With the support of the "check" function in the OC software, large-scale clinical trial dynamic management will be implemented to ensure that the data are collected completely, promptly, and accurately. The organizer will nominate monitors for regular visits to each unit for reexamination of the CRF to ensure consistency with the original data.

Ethical issues

Ethics statement

Researchers are responsible for ensuring that the study is conducted in accordance with the principles of the Declaration of Helsinki and GCP. Participants entirely voluntarily give their written informed consent before any study procedures, and they can voluntarily withdraw from the study for any reason. Parents or guardians are informed of the risks and benefits of the study if the participants have difficulty with decision-making. Each patient will be identified with a unique random number, and the private data must be preserved by researchers to maintain confidentiality.

Ethical approval

The study protocol, informed consent form (ICF), and other research documents were approved by the medical ethics committee of Zhongshan Hospital, Fudan University [approval registration number 2011-38(3)]. Other participating agencies were approved by the medical ethics committee of each hospital. (The names of all ethics committees that approved our study in every center are listed in Table 1.)

Clinical trial insurance

Before the trial, "safe clinical trial insurance" was purchased from Ping An Insurance Group (China) (policy number 10330001900105798721).

Informed consent form

The ICF must be signed by the participants or their representatives, and the date must be included. The signed ICF will be preserved by researchers and participants independently. The ICF preserved by the researchers will be made available to project managers for monitoring, auditing, and inspection.

Discussion

XBJ is a confidential National Technology Product approved by the Ministry of National Science and Technology and the State Secrecy Bureau, and is authorized as a patented product by the State Intellectual Property Office (patent protection period of 20 years). In 2004, XBJ was approved for marketing by the SFDA (number Z20040033). Preliminary clinical trial [36] results indicated that routine medication combined with XBJ can significantly reduce the death rate due to SP, and can also decrease the duration of mechanical ventilation and antibiotic use, with no adverse reactions such as liver and kidney dysfunction.

Blinding methods

Due to the characteristically colored liquid resulting from the TCM injection production process [37], it is difficult to produce a placebo with a similar appearance; therefore, most previous clinical trials have not been blinded in design. Such unblinded trials are subject to risks of selection bias and evaluation bias. To overcome this issue, we used photophobic infusion sets to implement a blinded design for the present study. Furthermore, this trial was designed with the aid of computer technology, using an updated version of the conventional processes of the central IWRS randomization system to conceal group information. Investigators and drug administrators will be given independent authority to log into this system following signing of a confidentiality agreement. All these methods were used to ensure

that the blinded design is implemented and maintained for the duration of the present study.

Strict randomization was implemented to minimize selection bias and evaluation bias. Furthermore, the use of the photophobic infusion set not only ensured that the blinded design of this trial was maintained but also avoided the placebo effect, rendering the research findings fair and objective. To our knowledge, the blinding and randomization methods used are being adopted in TCM injection clinical studies for the first time. These strategies can be used to overcome the difficulties associated with the blinded design of such studies, and also to investigate feasible approaches to clinical evaluation of other colored agents.

Outcome measures

In this trial, we selected improvement in PSI risk rating as the primary outcome. PSI risk rating is used to classify patients with pneumonia into 5 grades of increased risk for short-term mortality on the basis of 20 variables that are routinely available at presentation [38]. This risk rating precisely reflects the condition of patients with pneumonia [38]. It has been extensively validated in prospective and retrospective studies and is widely used in hospitals [39–41]. The secondary outcomes of this trial include several severity-of-illness scoring systems, such as LIS, MODS, and APACHE II, which reflect the condition of patients with SP from different perspectives and are predictive of patient mortality. Most previously reported SP trials used mortality or length of ICU stay as primary outcomes. However, many types of drugs and devices are used to treat patients in ICUs; therefore, it is difficult to establish a significant impact of a novel therapy on mortality among ICU patients. Consequently, in this study, selected indicators were used to reflect the condition of patients and to determine the significance of the therapy.

Safety of XBJ injection

The TCM injection is a new formulation of Chinese herbs based on a modification of the oral delivery of the traditional decoction. This alternative is characterized by rapid and efficient delivery and provides more choice for the clinical treatment of disease. However, there is little experience with TCM injections in the clinic; consequently, the safety of this formulation remains to be confirmed, and incorrect administration may be associated with AEs. Therefore, TCM injections must be used cautiously. Confirmation of the safety of XBJ injection as a TCM is of paramount importance. A meta-analysis of the efficacy and safety of XBJ injection [42] revealed that no AEs occurred in 1022 patients who received this treatment, indicating that XBJ injection is safe. There are also some reports of the AEs associated with XBJ injection [43], the majority of which occurred in patients receiving XBJ for the first time. Of the instances of AEs, 58.8 % occurred within 30 minutes of the injection, 64.7 % were caused by improper use of XBJ, and 47.1 % were recorded among patients with a history of allergies; all the patients improved after drug withdrawal.

In this trial, we also selected inflammation and coagulation index as well as hepatic and renal function as safety outcomes. All of these indicators will be closely monitored throughout the trial to determine the long-term safety of XBJ.

Strengths and limitations

This is a first large-sample, multicenter, blind RCT of TCM injection and was designed on the basis of previous studies. We adopted the central randomization system, electronic CRF, and other advanced systems and achieved innovative breakthroughs in the blinded design. The high standards for selection of the participating institutions and researchers, rigorous training of researchers, and the implementation process for inspection and other quality control methods were designed to ensure the quality of this trial. Ethical approval, signed informed consent, and clinical trial insurance coverage fully protect the interests of the subjects. We selected several severity-of-illness scoring systems as primary and secondary outcomes for objective evaluation the effects of XBJ. Throughout the trial, safety outcomes will be closely monitored to avoid AEs and crossover tables will be used to describe the changes in laboratory and electrocardiogram indicators. However, due to the large sample, the time of participant recruitment is long. The season and climate change may influence the prognosis of disease. The results of the trial remain to be confirmed in clinical research practice.

Trial status

Currently, participant recruitment is ongoing.

Abbreviations

AE: adverse event; APACHE: Acute Physiology and Chronic Health Evaluation; CRA: clinical research associate; CRF: case report form; CT: computed tomography; GCP: Good Clinical Practice; ICF: informed consent form; ICU: intensive care unit; IWRS: interactive web response system; LIS: Lung Injury Score; MODS: multiple organ dysfunction syndrome; OC: Oracle Clinical software; PSI: Pneumonia Severity Index; RCT: randomized clinical trial; SFDA: State Food and Drug Administration; SIRS: systemic inflammatory response syndrome; SP: severe pneumonia; TCM: traditional Chinese medicine; XBJ: Xuebijing.

Competing interests

The authors declare that they have no competing interests.

Authors' contributions

HCS sponsored the study and designed this protocol. CXB is the primary supervisor and participated in the design of this protocol. PW drafted the manuscript. YLS helped PW draft and revise the manuscript. HW, WKZ and MR participated in the design of the protocol and are responsible for trial management. ZL, JBZ and CY are involved in the data collection and

monitoring of the study. SL and ZQF are supervising this study and participated in revising the manuscript. All authors read and approved the final manuscript.

Acknowledgments
This work was supported by Tianjin University "Innovation Team Training Program" (Clinical Evaluation of Traditional Chinese Medicine Innovation Team [TD12-5032]) and Tianjin Chase Sun Pharmaceutical Co., Ltd.

Author details
[1]Tianjin University of Traditional Chinese Medicine, 312 Anshanxi Road, Nankai District, Tianjin 300193, China. [2]Department of Pulmonary Medicine, Zhongshan Hospital, Fudan University, Shanghai Institute of Respiratory Medicine, Shanghai 200032, China. [3]Tianjin Chase Sun Pharmaceutical Co. Ltd, 20 Quanfa Road, Tianjin Wuqing Development Area, Tianjin 300170, China. [4]Peking University Clinical Research Institute, 38 Xueyuan Road, Haidian District, Beijing 100191, China. [5]Key Laboratory of Chinese Internal Medicine of Ministry of Education and Beijing, Dongzhimen Hospital, Beijing University of Chinese Medicine, Haiyuncang Lane, Dongcheng District, Beijing 100700, China.

Received: 10 November 2015 Accepted: 5 March 2016
Published online: 17 March 2016

References

1. Sirvent JM, de la Torre MC, Lorencio C, Taché A, Ferri C, Garcia-Gil J, et al. Predictive factors of mortality in severe community-acquired pneumonia: a model with data on the first 24 h of ICU admission. Med Intensiva. 2013;37:308–15.
2. Feikin DR, Schuchat A, Kolczak M, Barrett NL, Harrison LH, Lefkowitz L, et al. Mortality from invasive pneumococcal pneumonia in the era of antibiotic resistance, 1995-1997. Am J Public Health. 2000;90:223–9.
3. Niederman MS, Mandell LA, Anzueto A, Bass JB, Broughton WA, Campbell GD, et al. Guidelines for the management of adults with community-acquired pneumonia: diagnosis, assessment of severity, antimicrobial therapy, and prevention. Am J Respir Crit Care Med. 2001;163:1730–54.
4. Restrepo MI, Mortensen EM, Velez JA, Frei C, Anzueto A. A comparative study of community-acquired pneumonia patients admitted to the ward and the ICU. Chest. 2008;133:610–7.
5. World Health Organization (WHO). The top 10 causes of death. Fact Sheet 310 [updated May 2014]. http://www.who.int/mediacentre/factsheets/fs310/en/. Accessed 12 March 2016.
6. Hagiwara S, Iwasaka H, Hasegawa A, Hidaka S, Uno A, Ueo K, et al. Filtration leukocytapheresis therapy ameliorates lipopolysaccharide-induced systemic inflammation in a rat model. J Surg Res. 2011;171:777–82.
7. Matsuse H, Yanagihara K, Mukae H, Tanaka K, Nakazato M, Kohno S. Association of plasma neutrophil elastase levels with other inflammatory mediators and clinical features in adult patients with moderate and severe pneumonia. Respir Med. 2007;101:1521–8.
8. Levi M, van der Poll T, Schultz M. New insights into pathways that determine the link between infection and thrombosis. Neth J Med. 2012;70:114–20.
9. De Pascale G, Bello G, Tumbarello M, Antonelli M. Severe pneumonia in intensive care: cause, diagnosis, treatment and management: a review of the literature. Curr Opin Pulm Med. 2012;18:213–21.
10. Ariani F, Liu K, Jing Z, Qu J. Glucocorticosteroid in treatment of severe pneumonia. Mediators Inflamm. 2013;2013:865635. doi:10.1155/2013/865635.
11. Ulloa L, Tracey KJ. The "cytokine profile": a code for sepsis. Trends Mol Med. 2005;11:56–63.
12. Torres-Rosas R, Yehia G, Peña G, Mishra P, del Rocio T-BM, Moreno-Eutimio MA, et al. Dopamine mediates the vagal modulation of the immune system by electroacupuncture. Nat Med. 2014;20:291–5.
13. Wang Y, Ji M, Wang L, Chen L, Li J. Xuebijing injection improves the respiratory function in rabbits with oleic acid-induced acute lung injury by inhibiting IL-6 expression and promoting IL-10 expression at the protein and mRNA levels. Exp Ther Med. 2014;8:1593–8.
14. Wang J. Integrative treatment of acute critical illness diagnosis and treatment ideas and practice course. Tianjin J Tradit Chin Med. 1998;15:241–2.
15. Xue L. Research of the new strategy for the treatment of systemic inflammatory response syndrome and multiple organ dysfunction syndrome: an experimental study of the effect of traditional Chinese herb "xue bi jing.". Chin Crit Care Med. 1997;9:18–20. 64.
16. Jiao L, Yao Y, Shou S. Effects of Xuebijing injection on hepatic gelsolin and inflammatory cytokine in septic rats. Med J Chin Peoples Liberation Army. 2011;36:14–6.
17. He XD, Wang Y, Wu Q, Wang HX, Chen ZD, Zheng RS, et al. Xuebijing protects rats from sepsis challenged with *Acinetobacter baumannii* by promoting annexin A1 expression and inhibiting proinflammatory cytokines secretion. Evid Based Complement Alternat Med. 2013;2013:804940. doi:10.1155/2013/804940.
18. Geng P, Xia P, Gu J, Xu M, Xu J, Tan D, et al. Intervention of Xuebijing injection against early hypercoagulability in patients with severe sepsis. Chin J Integr Tradit West Med Intensive Crit Care. 2008;15:346–9.
19. Dai XG, Yao YM, Ai YH, Yu Y. The enhancing effect of "Xuebijing injection" on lipopolysaccharide-induced apoptosis of regulatory T cells and mediation of polarization of helper T cells [in Chinese]. Zhonghua Shao Shang Za Zhi. 2009;25:106–10.
20. Zhu MJ, Zhang G, Hu MH, Chen YB, Ji CL. Stasis-resolving and detoxifying effect of Xuebijing injection on severe pneumonia: a systematic review. Chin J Evid Based Med. 2014;14:462–8.
21. Mandell LA, Wunderink RG, Anzueto A, Bartlett JG, Campbell GD, Dean NC, et al. Infectious Diseases Society of America/American Thoracic Society consensus guidelines on the management of community-acquired pneumonia in adults. Clin Infect Dis. 2007;44 Suppl 2:S27–72.
22. Bone RC, Balk RA, Cerra FB, Dellinger RP, Fein AM, Knaus WA, et al. Definitions for sepsis and organ failure and guidelines for the use of innovative therapies in sepsis. Chest. 1992;101:1644–55.
23. The ARDS Definition Task Force. Acute respiratory distress syndrome: the Berlin Definition. JAMA. 2012;307:2526–33.
24. Dellinger RP, Levy MM, Rhodes A, Annane D, Gerlach H, Opal SM, et al. Surviving Sepsis Campaign: international guidelines for management of severe sepsis and septic shock, 2012. Intensive Care Med. 2013;39:165–228.
25. Julián-Jiménez A, Reyes MJ P de l, Parejo Miguez R, Laín-Terés N, Cuena-Boy R, Lozano-Ancín A. Improved management of community-acquired pneumonia in the emergency department. Arch Bronconeumol. 2013;49:230–40.
26. Li J, Qiao Y, Zhang H, et al. The therapeutic effects of Xuebijing injection on community acquired pneumonia patients accompanied by acute kidney injury. Chin J Integr Tradit West Med Intensive Crit Care. 2012;19(4):226–9.
27. Woodhead M, Blasi F, Ewig S, Huchon G, Ieven M, Ortqvist A, et al. Guidelines for the management of adult lower respiratory tract infections. Eur Respir J. 2005;26:1138–80.
28. Gustot T, Durand F, Lebrec D, Vincent JL, Moreau R. Severe sepsis in cirrhosis. Hepatology. 2009;50:2022–33.
29. Murray JF, Matthay MA, Luce JM, Flick MR. An expanded definition of the adult respiratory distress syndrome. Am Rev Respir Dis. 1988;138:720–3.
30. Marshall JC, Cook DJ, Christou NV, Bernard GR, Sprung CL, Sibbald WJ. Multiple organ dysfunction score: a reliable descriptor of a complex clinical outcome. Crit Care Med. 1995;23:1638–52.
31. Middleton PM. Practical use of the Glasgow Coma Scale: a comprehensive narrative review of GCS methodology. Australas Emerg Nurs J. 2012;15:170–83.
32. Knaus WA, Draper EA, Wagner DP, Zimmerman JE. APACHE II: a severity of disease classification system. Crit Care Med. 1985;13:818–29.
33. Ferreira FL, Bota DP, Bross A, Mélot C, Vincent JL. Serial evaluation of the SOFA score to predict outcome in critically ill patients. JAMA. 2001;286:1754–8.
34. Oracle Corporation. Oracle Clinical. http://www.oracle.com/us/products/applications/health-sciences/e-clinical/clinical/index.html. Accessed 12 March 2016.
35. Shang HC, Zhang JH, Dai GH, Cao HB, Ren M, Xiang YZ, et al. Administration and quality control of large-scale clinical trials of traditional Chinese medicine [in Chinese]. Zhong Xi Yi Jie He Xue Bao. 2007;5:1–4.
36. Song Y, Song Z, Jiang J, Bai C. Clinical efficacy and safety evaluation of Xuebijing on severe pneumonia. Int J Respir. 2012;32(22):1692–5.
37. Food S, Administration D. National Drug Standards (amendment) promulgated pieces. Drug Stand China. 2013;14:448–73.
38. Renaud B, Coma E, Labarere J, Hayon J, Roy PM, Boureaux H, et al. Routine use of the Pneumonia Severity Index for guiding the site-of-treatment decision of patients with pneumonia in the emergency department: a multicenter, prospective, observational, controlled cohort study. Clin Infect Dis. 2007;44:41–9.
39. Fine MJ, Auble TE, Yealy DM, Hanusa BH, Weissfeld LA, Singer DE, et al. A prediction rule to identify low-risk patients with community-acquired pneumonia. N Engl J Med. 1997;336:243–50.

40. Marras TK, Gutierrez C, Chan CK. Applying a prediction rule to identify low-risk patients with community-acquired pneumonia. Chest. 2000;118:1339–43.
41. Arnold FW, Ramirez JA, McDonald LC, Xia EL. Hospitalization for community-acquired pneumonia: the pneumonia severity index vs clinical judgment. Chest. 2003;124:121–4.
42. Hu J, Shang H, Li J, Zhang J, Zhang L, Zhang B. Xuebijing injection for sepsis: a comprehensive review. Med J Chin Peoples Liberation Army. 2010;35:9–12.
43. Xie F, Huang Q. Literature analysis of adverse reactions of Xuebijing injection [in Chinese]. Strait Pharm J. 2013;1:283–4.

Submit your next manuscript to BioMed Central and we will help you at every step:

- We accept pre-submission inquiries
- Our selector tool helps you to find the most relevant journal
- We provide round the clock customer support
- Convenient online submission
- Thorough peer review
- Inclusion in PubMed and all major indexing services
- Maximum visibility for your research

Submit your manuscript at
www.biomedcentral.com/submit

BioMed Central
